# Capsaicin induces ferroptosis via suppression of SLC7A11 activity and upregulation of ACSL4 mediated by AMPK in tongue squamous cell carcinoma

**DOI:** 10.3389/fonc.2025.1532555

**Published:** 2025-05-14

**Authors:** Qiwei Zhao, Yu Wang, Long Ding, Zhuang Li, Mengyang Wang, Yueqing Huang, Qiushi Cao, Yaqin Sun, Xiaohong Guo

**Affiliations:** ^1^ Department of Basic Medicine, Hubei University of Chinese Medicine, Wuhan, China; ^2^ Hubei Shizhen Laboratory, Wuhan, China

**Keywords:** capsaicin, ferroptosis, tongue squamous cell carcinoma, AMPK, ACSL4, SLC7A11

## Abstract

**Introduction:**

The global incidence of tongue squamous cell carcinoma (TSCC) has been steadily increasing. Our previous studies have demonstrated that capsaicin (CAP) promotes apoptosis and inhibits cell migration, thereby exerting anti-TSCC effects. In this study, we aimed to validate whether CAP induces ferroptosis in TSCC and to elucidate the underlying mechanisms.

**Methods:**

Cell viability in HN6 and CAL27 cells was assessed using CCK-8 assays. Mitochondrial structural changes were observed via transmission electron microscopy (TEM). The levels of malondialdehyde (MDA), Fe^2+^, reactive oxygen species (ROS), and glutathione (GSH) were measured by the corresponding assay kits. Ferrostatin-1 (Fer-1) was utilized to confirm the involvement of ferroptosis. Western blotting was employed to evaluate the phosphorylation of AMP-activated protein kinase (AMPK), acyl-CoA synthetase long-chain family member 4 (ACSL4), and glutathione peroxidase 4 (GPX4). Additionally, Glutamic acid release was determined using an assay kit. The interaction between BECN1 and solute carrier family 7 member 11 (SLC7A11) was analyzed by co-immunoprecipitation (Co-IP). To elucidate the underlying mechanisms, lentiviral-mediated shRNA knockdown of AMPK was performed, with subsequent *in vivo* validation.

**Results:**

CAP significantly suppressed the viability of HN6 and CAL27 cells. TEM analysis revealed mitochondrial damage following CAP treatment. Furthermore, CAP increased levels of MDA, Fe²⁺, and ROS while decreasing GSH; these alterations were reversed by Fer-1 treatment. Western blot analyses indicated that CAP upregulated phosphorylated AMPK and ACSL4 but downregulated GPX4 expression. Moreover, CAP inhibited glutamate release while enhancing BECN1-SLC7A11 binding, suggesting a reduction in SLC7A11 activity through the AMPK/BECN1 pathway. Notably, AMPK inhibition mitigated CAP-induced changes in p-BECN1, ACSL4, MDA, Fe²⁺, GSH, and ROS levels. *In vivo* experiments corroborated these findings.

**Discussion:**

Our study demonstrates that CAP activate the AMPK signaling, inhibits the activity of SLC7A11 and increases ACSL4 expression, thereby inducing ferroptosis in TSCC. These findings, supported by *in vivo* data, highlight CAP’s role in triggering ferroptosis as an anti-TSCC mechanism.

## 1 Introduction

According to the International Agency for Research on Cancer, in 2022, there were over 380,000 new cases of oral cancer reported globally, with more than 90% classified as squamous cell carcinoma (OSCC). During the same period, approximately 180,000 deaths were recorded (1). OSCC primarily includes cancers of the lip, gum, tongue, jawbone, floor of the mouth, and oropharynx. Among of which, tongue squamous cell carcinoma (TSCC) is the most common type, accounting for about 41% (2). This malignancy arises due to the anatomical susceptibility of the tongue to carcinogen exposure and its propensity for early lymphatic metastasis. Clinically aggressive in nature, TSCC often presents with nodal involvement that necessitates radical dissection; however, extensive resections can lead to significant deficits in speech and swallowing functions (3). Currently, chemotherapy remains an important adjunctive method to control distant metastasis of TSCC and improve prognosis. However, chemoresistance is one of the reasons for the lack of significant improvement in survival rates (4). Key mechanisms contributing to drug resistance encompass enhanced cellular detoxification, inhibition of apoptosis, dysregulation of DNA repair processes, and the involvement of non-coding RNAs (5).Therefore, reducing toxic side effects of chemotherapeutic drugs and overcoming tumor cell resistance are urgent problems to be solved in clinical diagnosis and treatment. The development of new drugs or drug repurposing has become a major research direction in the treatment of TSCC.

Capsaicin (CAP) is the primary spicy component found in chili peppers that are commonly consumed on a daily basis. It has functions such as antioxidation and lipid-lowering. Its traditional biological roles mainly include treating neuropathic pain, hyperalgesia, and inflammation (6). In recent years, numerous studies have demonstrated that CAP could induce cell cycle arrest, apoptosis, and autophagy in tumor cells or inhibit their metabolic activation. For example, Chen et al. observed that CAP could inhibit the stemness of osteosarcoma and suppress its migratory ability by downregulating SOX2 and EZH2 (7). Xiao et al. discovered that capsaicin inhibited the proliferation of breast cancer by downregulating the NF-κB pathway mediated by FBI-1 and promoting apoptosis (8). Lin et al. demonstrated that capsaicin suppresses the growth of bladder cancer cells by inhibiting tNOX and SIRT1, thereby reducing cell proliferation, inhibiting cell migration, and prolonging the cell cycle progression (9). Our previous studies have shown that CAP exerts pro-apoptotic effects in TSCC through the calpain pathway mediated by transient receptor potential vanilloid 1 (TRPV1) (10). Here, we sought to further explore the potential of CAP in inhibiting the growth of TSCC through alternative mechanisms.

AMP-activated protein kinase (AMPK) serves as a crucial cellular energy sensor and metabolic regulator, functioning as the “master switch” for maintaining cellular energy homeostasis (11). The activation of AMPK modulates various metabolic processes to enhance energy production while concurrently inhibiting high-energy-consuming activities such as protein synthesis, fatty acid synthesis, and glycogen synthesis, thereby conserving cellular energy (12). Current research has established that AMPK exerts an inhibitory effect on tumors (13–15). Our previous studies have demonstrated that CAP activates AMPK, inhibits epithelial-mesenchymal transition (EMT) and migration in TSCC, and reverses the resistance of TSCC to cisplatin, ultimately resulting in the reduction of tumor growth (16, 17).

Ferroptosis, a form of cell death first reported in 2012, is distinct from apoptosis, necrosis, autophagy, and other forms of cell death. It is characterized by unique morphological, biochemical, genetic and immunological features (18). Current research has linked the physiological functions of ferroptosis to various human diseases, such as cancer, diabetes and osteoarthritis, with its core pathogenic mechanisms primarily involving iron overload and lipid peroxidation (LPO), as well as mitochondrial dysfunction (19–21). Key regulators of ferroptosis include ACSL4, system X_c_^-^, GPX4 and so on. Among of which, ACSL4 serves as a crucial enzyme in fatty acid metabolism. It activates and incorporates polyunsaturated fatty acids (PUFAs) into cell membranes, facilitates LPO, and ultimately contributes to cellular damage and death (22). System X_c_^-^, consisting of SLC7A11 and SLC3A2, is an amino acid transporter located on the cell membrane that operates on a 1:1 exchange mechanism, transporting cystine into the cell and glutamate out. It influences the synthesis of glutathione (GSH), a potent antioxidant that reduces the formation of lipid peroxides and plays a key role in ferroptosis (23). Severe lipid peroxidation can directly induce ferroptosis, while GPX4 utilizes GSH as a reductant to reduce lipid peroxides. This action effectively prevents the accumulation of harmful substances and mitigates damage to the cell membrane (24). Further research has found that AMPK-mediated phosphorylation of BECN1 at serine residues enhances its interaction with SLC7A11, resulting in the inhibition of SLC7A11 activity. This process ultimately facilitates ferroptosis (25).

Prior evidence has demonstrated that CAP can induce iron-dependent cell death in various cancer types, such as non-small cell lung cancer (NSCLC) (26) and osteosarcoma (27). Our previous study revealed that capsaicin exerts pro-apoptotic effects in TSCC through the calpain pathway via TRPV1 (10). Notably, TRPV1-mediated Ca²^+^ influx could also directly participates in ferroptosis (27), indicating an interconnected molecular crosstalk between ferroptosis and apoptosis. However, the complete elucidation of whether CAP induces ferroptosis remains to be achieved, necessitating further investigation to understand the specific mechanisms involved. Therefore, the objective of this study was to examine the effects of CAP on ferroptosis in TSCC and to explore the regulatory role of AMPK in this process, thereby providing a theoretical basis for the clinical application of CAP in treating TSCC.

## 2 Materials and methods

### 2.1 Cell culture and reagents

HN6 cells were provided by the Affiliated Stomatological Hospital of Shanxi Medical University. CAL27 cells were purchased from the American Type Culture Collection (ATCC), and 293T cells were obtained from Wuhan BioEagle Biological Science & Technology Co., Ltd. All cell lines were cultured in Dulbecco’s Modified Eagle’s Medium (DMEM) (BL301A, Biosharp) containing 1% penicillin-streptomycin solution (BL505A, Biosharp) and 10% fetal bovine serum (FBS) (FSP500, ExCell), and maintained at 37°C in an incubator with 5% CO_2_.

Capsaicin (HY-10448) and Ferrostatin-1 (HY-100579) were purchased from MedChemExpress (Shanghai, China). The antibodies included ACSL4 (22401-1-AP, Proteintech, 1:8000), GPX4 (30388-1-AP, Proteintech, 1:1000), SLC7A11 (A2413, ABclonal, 1:1000), BECN1 (11306-1-AP, Proteintech, 1:1000), p-BECN1 (Ser90, AP1254, ABclonal, 1:1000), AMPK (18167-1-AP, Proteintech, 1:1000), p-AMPK (Thr172, AF3423, Affinity, 1:1000), β-actin as an internal control (AC038, ABclonal, 1:10000) and HRP-conjugated goat anti-rabbit IgG secondary antibody (BS13278, Bioworld, 1:100000).

### 2.2 Cell counting Kit-8 assay

HN6 and CAL27 cells were seeded in 96-well plates at a density of 1 × 10^3^ cells per well and cultured until reaching 70% confluence. After treatment with CAP at a concentration of 150 μM for 24 hours, 10 μL Cell Counting Kit-8 (CCK-8) reagent (28) (MA0218, Dalian Meilun Biology Technology Co. Ltd) was added to each well. The optical density (OD) at 450 nm was measured using a microplate reader (MR-96A, Shenzhen Mindray) to assess cell viability.

### 2.3 Transmission electron microscopy (TEM)

HN6 and CAL27 cells were initially cultured at a density of 5 × 10^6^ cells in T25 flasks. Once the cells adhered, they were treated with 150 μM CAP for 24 hours. then the cells were digested and collected, fixed with 2.5% glutaraldehyde at 4°C for 6 hours. Following fixation, the cells were dehydrated through a graded series of ethanol and acetone solutions. Subsequently, the dehydrated samples were embedded in resin and sectioned. The sections were stained with a solution of 3% uranyl acetate and 0.4% lead citrate before being examined using a transmission electron microscope (10) (JEM-1400 Plus, JEOL, Japan).

### 2.4 Malondialdehyde (MDA) assay

HN6 and CAL27 cells were seeded in 6-well plates at a density of 1×10^6^ per well, followed by treatment with 150 μM CAP, with or without 1 μM Fer-1. MDA levels were measured according to the instructions of the MDA Assay Kit (29) (RXWB0005-96, Ruixin Biotech), and the absorbance was read at 532 nm and 600 nm using a microplate reader (MR-96A, Shenzhen Mindray).

### 2.5 Fe^2+^ assay

HN6 and CAL27 cells were seeded in 6-well plates at a density of 1×10^6^ per well. Subsequently, they were treated with 150 μM CAP, with or without 1 μM Fer-1. The levels of Fe^2+^ were measured according to the instructions of the Fe^2+^ Assay Kit (26) (RXWB0464-96, Ruixin Biotech), and the absorbance was recorded at 562 nm using a microplate reader (MR-96A, Shenzhen Mindray).

### 2.6 Glutathione (GSH) assay

HN6 and CAL27 cells were seeded in 6-well plates at a density of 1×10^6^ per well. Then they were treated with 150 μM CAP, with or without 1 μM Fer-1. GSH levels were detected following the instruction of the GSH Assay Kit (30) (A006-2-1, Nangjing Jiancheng Bioengineering). The absorbance was read at 405 nm using a microplate reader (MR-96A, Shenzhen Mindray).

### 2.7 Detection of reactive oxygen species

HN6 and CAL27 cells were inoculated into 6-well plates at a density of 1×10^6^ per well and treated with 150 μM CAP for 24 h, with or without 1 μM Fer-1. After that, the 2’,7’-Dichlorodihydrofluorescein diacetate (31)(DCFH-DA) (S0033S, Beyotime Biotechnology) was added and incubated at 37°C for 30 min, then observed under a fluorescence microscope (MR-96A, Shenzhen Mindray).

### 2.8 Glutamate release assay

Glutamate release levels were detected following the instruction of the Glutamate Content Assay Kit (32) (BC5215, Solarbio). The absorbance was read at 450 nm using a microplate reader (MR-96A, Shenzhen Mindray).

### 2.9 Co-immunoprecipitation

The cultured HN6 and CAL27 cells were harvested and lysed to obtain total protein. The immunoprecipitation procedure was carried out according to the kit instructions (33) (P2179S, Beyotime Biotechnology), using specific antibodies and protein lysates. The precipitated proteins were then collected using protein A/G magnetic beads, followed by thorough washing of the beads. Subsequently, the proteins were eluted by boiling in SDS sample buffer, and Western blot analysis was performed.

### 2.10 Stable transfection

The lentiviral vector pLenti-shRNA-GFP-Puro, along with the packaging plasmids PSPAX-2 and PMD2G, were co-transfected into 293T cells. The viral supernatant was collected and filtered through a 0.22 μm filter for subsequent infection. HN6 cells were seeded in a 6-well plate and cultured for 24 hours before viral infection. After 72 hours post-infection, puromycin was added to select infected cells. The knockdown efficiency of AMPK was then confirmed by Western blot analysis (17). The sequence of shRNA was as follow: 5’-GGA AGT TCT CAG CTG TCT TTA-3’.

### 2.11 Western blot analysis

HN6 and CAL27 cells were cultured in a 6-well plate and treated with CAP at a concentration of 150 μM. Protein extraction was carried out using RIPA lysis buffer (BL504A, Biosharp) followed by determination of protein concentration using a BCA kit (20201ES76, Yeasen). Subsequently, 20 μg protein samples were separated by SDS-PAGE and transferred to PVDF membranes (IPVH00010, Millipore). The membranes were incubated in 5% non-fat milk at room temperature for 1.5 hours, and then incubated with primary antibody overnight at 4°C, followed by the second antibody for 1 hour at room temperature. The ECL chemiluminescent substrate (BL523A, Biosharp) was then applied onto the membrane (34). Subsequently, the protein bands were observed in a gel imaging system (ChemiDoc XRS+; Bio-Rad Laboratories, Inc.) and finally analyzed with Image J.

### 2.12 *In vivo* studies

All male BALB/c nude mice (6 weeks old) were purchased from Zhuhai, Guangzhou and housed at the Experimental Animal Centre of Hubei University of Chinese Medicine. The temperature was maintained at 26°C-28°C, with relative humidity at 40%-60%, and a 12-hour light/dark cycle. After 1 week of acclimatization, the nude mice were injected subcutaneously with 5×10^6^ HN6 or HN6 (shAMPK) cells. Treatment was initiated when the tumor volume reached 100 mm^3^. Mice were randomly assigned to three experimental groups of five mice each: 1) Blank control group, administered 200 µL PBS by gavage; 2) CAP group, administered 10 mg/kg CAP dissolved in 200 µL PBS by gavage; 3) CAP+ shAMPK group, administered 10 mg/kg CAP dissolved in 200 µL PBS by gavage (10). Tumor size was measured every 3 days. At the conclusion of the study, the mice were sacrificed, and the excised tumors were collected and weighed. Livers and kidneys were subjected to fixation, embedding, and sectioning, followed by staining with hematoxylin and eosin (HE). Immunohistochemistry (IHC) was performed to evaluate the expression of p-BECN1(Ser90, AP1254, ABclonal, 1:100), ACSL4 (22401-1-AP, Proteintech, 1:100) and GPX4 (30388-1-AP, Proteintech, 1:100) in xenograft tumor tissues. Furthermore, the levels of MDA, Fe^2+^ and GSH in the tumor were assessed using the corresponding analysis kits mentioned above.

### 2.13 Ethics

The animal handling procedures employed in this research were conducted in accordance with the Guide for the Care and Use of Laboratory Animals established by the National Institutes of Health and were approved by the Institutional Review Board of Hubei University of Chinese Medicine (HUCMS24406389).

### 2.14 Statistical analysis

Data analysis was performed using GraphPad Prism software version 9.5. The results are presented as the mean ± SEM (Standard Error of Mean) from three independent experiments. Differences between groups were analyzed using one-way ANOVA, and comparisons between two groups were conducted using the t-test. *P* < 0.05 was considered statistically significant.

## 3 Results

### 3.1 Cell proliferation was inhibited by CAP through ferroptosis-dependent pathway in TSCC cells

**Figure 1:**
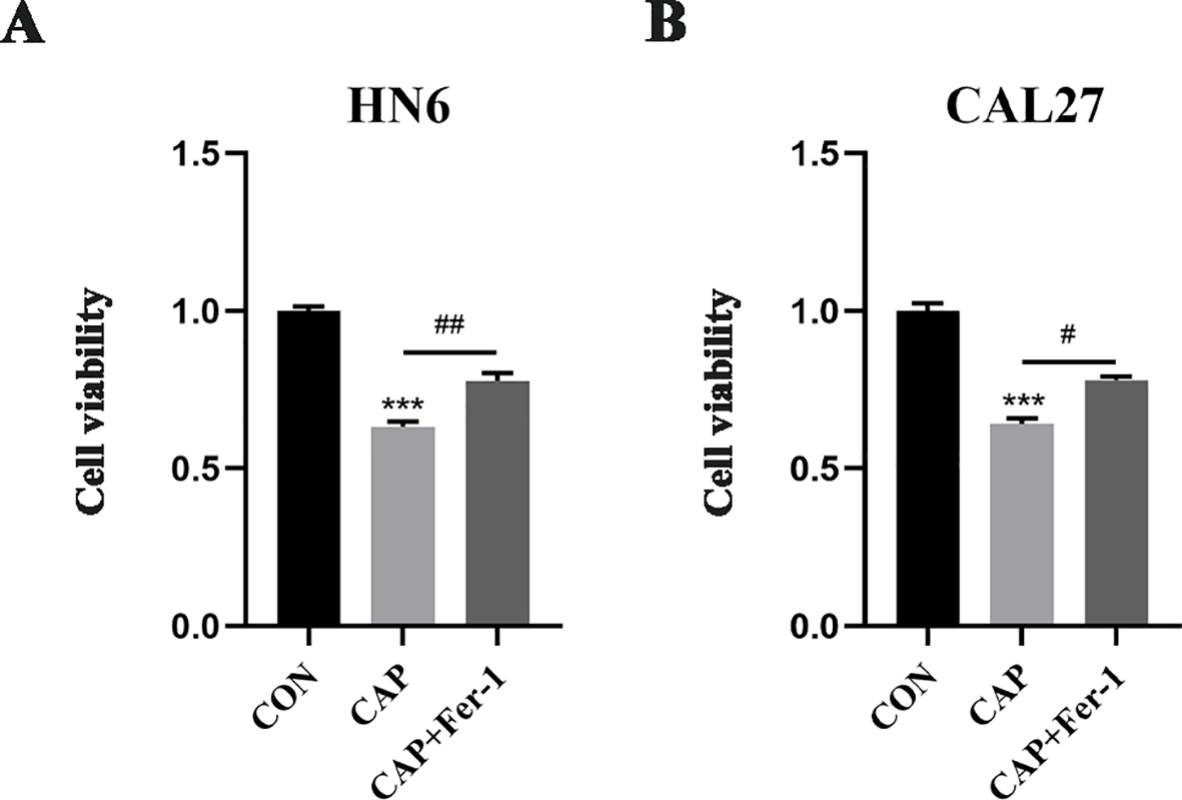
Cell proliferation was inhibited by CAP through ferroptosis-dependent pathway in TSCC cells. **(A, B)** HN6 and CAL27 cells were treated with 150 μM CAP and 1 μM Fer-1 for 24h, then cell viabilities were detected by CCK-8 assay. *** *P* < 0.001 vs. CON; ^#^ *P* < 0.05, ^##^ *P* < 0.01 vs. CAP. CON, control; CAP, capsaicin.

HN6 and CAL27 cells were initially pre-treated with 1 μM ferrostatin-1 (Fer-1), a ferroptosis inhibitor. Subsequently, the cells were exposed to 150 μM CAP for 24 hours. CCK-8 assay revealed that CAP significantly inhibited the cell viability in both HN6 and CAL27 cells, while the pre-treatment with Fer-1 partially reversed the effect of CAP (**Figures 1A, B**). These findings suggested that CAP could potentially inhibited the cell proliferation through ferroptosis-dependent pathway.

### 3.2 Ferroptosis was induced by CAP in TSCC cells

**Figure 2:**
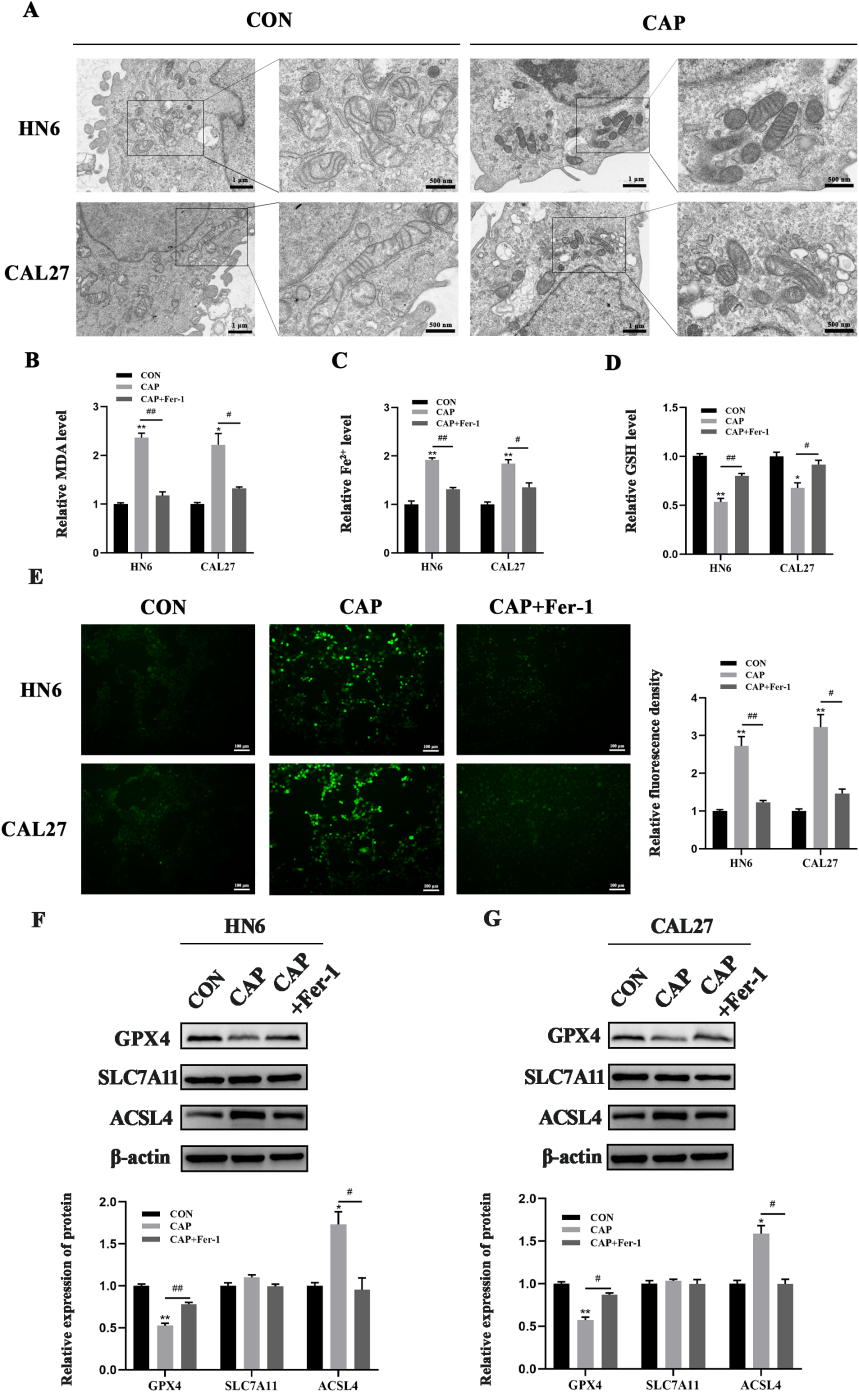
Ferroptosis was induced by CAP in TSCC cells. **(A)** HN6 and CAL27 cells were treated with CAP for 24 h, then the cells were observed by transmission electron microscopy. The images were magnified at a scale of ×8.0k, with localized magnification at ×20.0k. **(B–E)** MDA **(B)**, Fe^2+^ **(C)**, GSH **(D)** and ROS levels **(E)** of HN6 and CAL27 cells were measured after CAP treatment, with or without Fer-1. **(F, G)** Western blot analysis of GPX4, SLC7A11 and ACSL4 in HN6 and CAL27 cells. ^*^ *P* < 0.05, ^**^ *P* < 0.01 vs. CON; ^#^ *P* < 0.05, ^##^ *P* < 0.01 vs. CAP. CON, control; CAP, capsaicin.

When ferroptosis occurs, the cells undergoes the accumulation of MDA、Fe^2+^ and ROS, as well as a reduction in GSH levels. Additionally, ferroptosis leads to alterations in mitochondrial structure. Here, TEM results revealed that CAP caused mitochondrial shrinkage, membrane rupture, and cristae fragmentation in both cell lines (**Figure 2A**), which were consistent with the characteristics of ferroptosis in mitochondria (35). The level of lipid oxidation product MDA and Fe^2+^, which served as markers of ferroptosis, was found to be elevated by CAP treatment, while Fer-1 reversed these CAP-induced accumulation (**Figures 2B, C**). Conversely, CAP induced a significant reduction in GSH levels, which could be reversed by treatment with Fer-1 (**Figure 2D**). Following treatment with CAP, fluorescence microscopy results revealed an accumulation of ROS in HN6 and CAL27 cells (**Figure 2E**). Furthermore, GPX4, SLC7A11 and ACSL4 play crucial roles in regulating ferroptosis. Western blot analysis revealed that CAP led to a decrease in GPX4 protein levels and an increase in ACSL4 protein levels in both HN6 and CAL27 cells, though no significant effect was detected on the expression of SLC7A11 (**Figures 2F, G**). The results above provided credible evidences of ferroptosis induced by CAP in TSCC cells.

### 3.3 The activity of SLC7A11 was suppressed through AMPK/BECN1 pathway during the ferroptosis induced by CAP in TSCC cells

**Figure 3:**
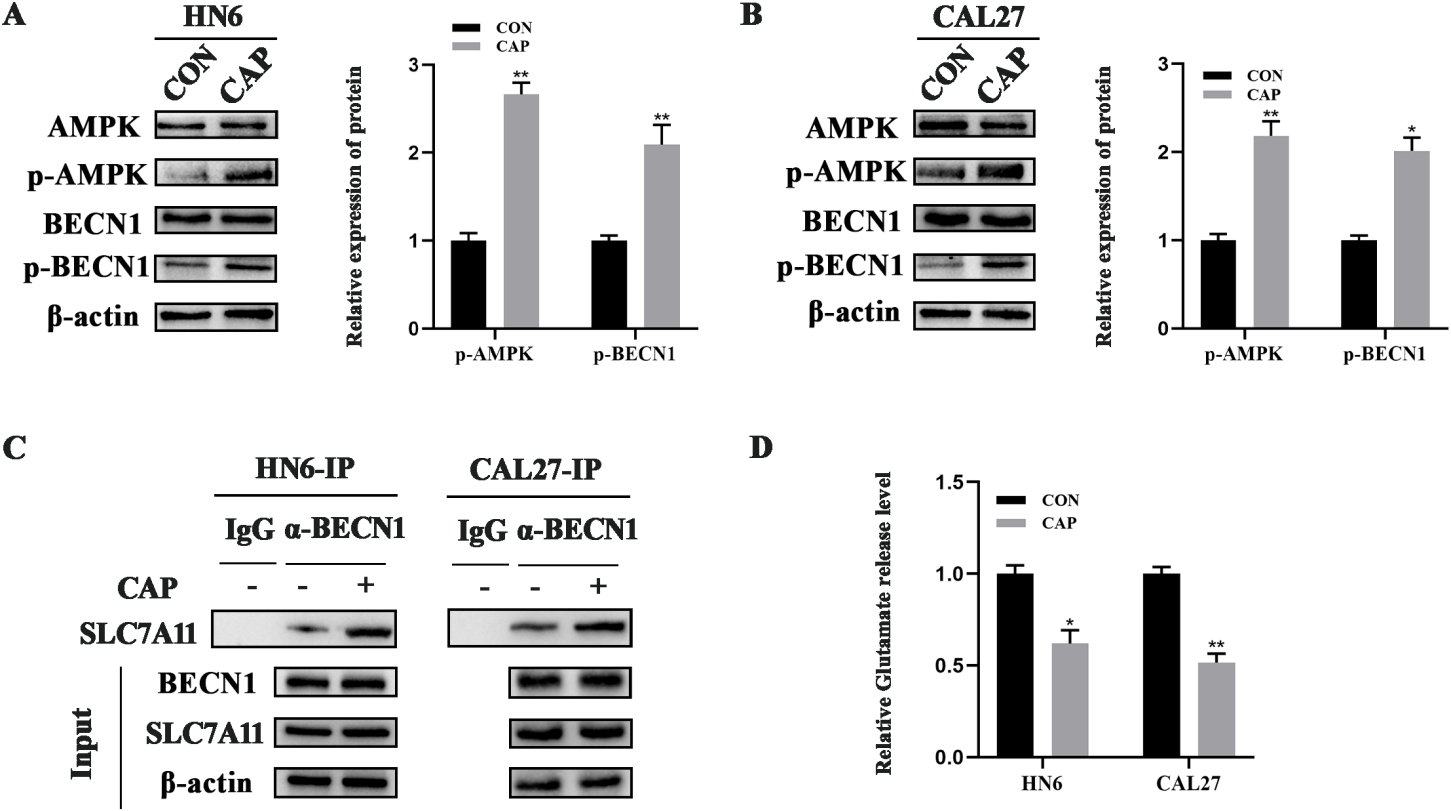
The activity of SLC7A11 was suppressed during the ferroptosis induced by CAP in TSCC cells. **(A, B)** Western blot analysis of AMPK/BECN1 axis in HN6 and CAL27 cells. **(C)** Co-immunoprecipitation analysis of the BECN1-SLC7A11 complex **(D)** Glutamate release level was measured after CAP treatment. * *P* < 0.05, ** *P* < 0.01 vs. CON. CON, control.

A previous study has demonstrated that AMPK can phosphorylate BECN1, leading to the formation of the BECN1-SLC7A11 complex, which inhibits SLC7A11 activity and promotes ferroptosis (25). To investigate the reason why the expression of SLC7A11 was unchanged after CAP treatment in HN6 and CAL27 cells, the AMPK/BECN1 signaling pathway was assessed and the results indicated that AMPK was significantly activated and the levels of phosphorylated BECN1 was increased remarkably by CAP (**Figures 3A, B**), which suggested the activation of AMPK/BECN1 signaling pathway. Furthermore, co-immunoprecipitation results revealed that CAP promoted the binding of BECN1 and SLC7A11 in HN6 and CAL27 cells (**Figure 3C**). Meanwhile, the activity of SLC7A11 was suppressed by CAP which was evidenced by a reduction in glutamate release levels (**Figure 3D**). These results suggested that the activity of SLC7A11 was suppressed through AMPK/BECN1 pathway during the ferroptosis induced by CAP in TSCC cells.

### 3.4 AMPK/BECN1 and AMPK/ACSL4 pathways were involved in the ferroptosis induced by CAP in TSCC cells

**Figure 4:**
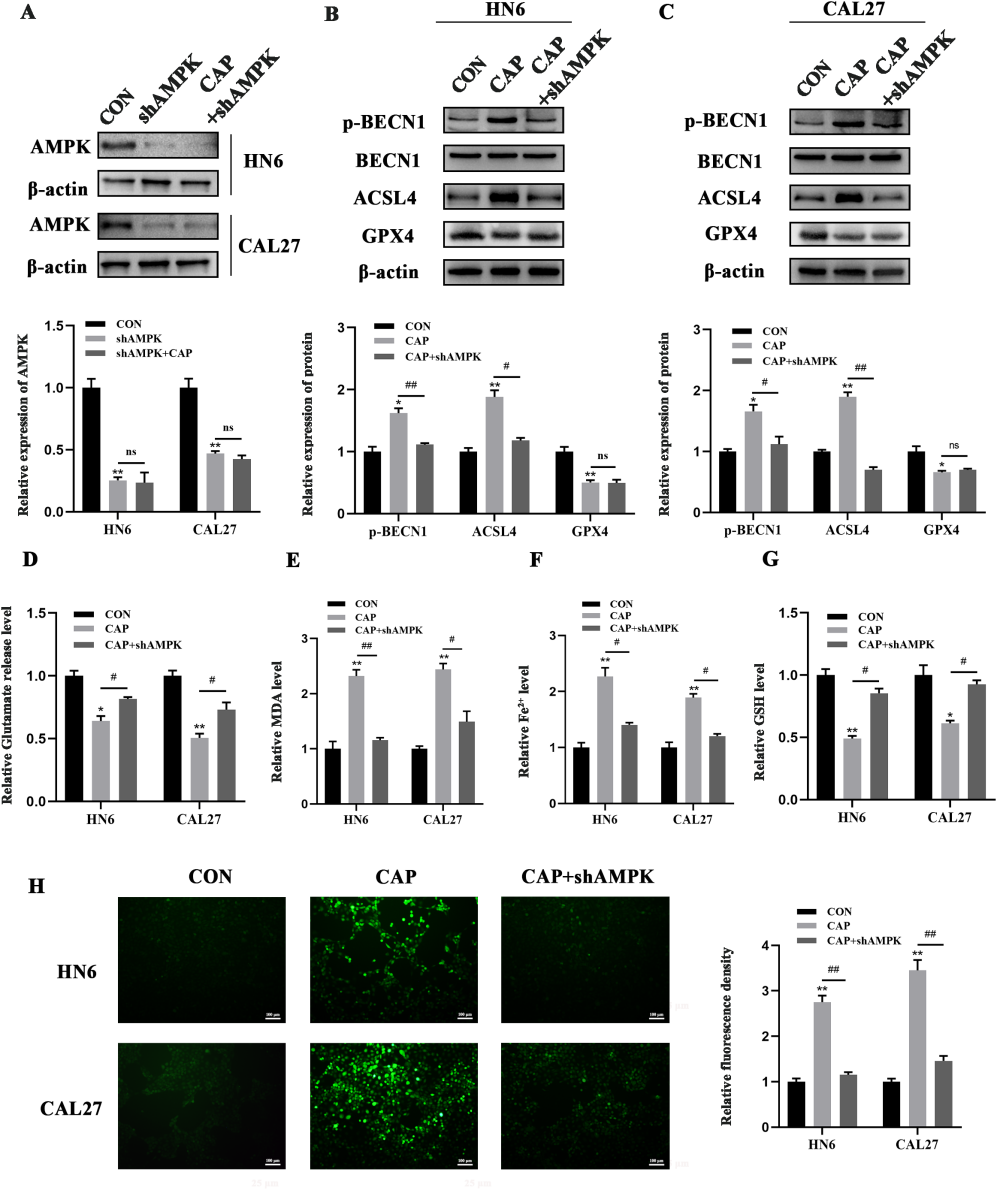
AMPK/BECN1 and AMPK/ACSL4 pathways were involved in the ferroptosis induced by CAP in TSCC cells. **(A)** Western blot analysis of AMPK knockdown efficiency. **(B, C)** Western blot analysis of p-BECN1, BECN1, ACSL4 and GPX4 after AMPK knockdown. **(D–G)** Glutamate release **(D)**, MDA **(E)**, Fe^2+^ **(F)**, GSH **(G)** and ROS levels **(H)** of HN6 and CAL27 cells were measured after CAP treatment, with or without AMPK knockdown. ns, no significance; * *P* < 0.05, ** *P* < 0.01 vs. CON; ^#^ *P* < 0.05, ^##^ *P* < 0.01 vs. CAP. CON, control; CAP, capsaicin.

ACSL4 is known to trigger ferroptosis within lipid metabolism pathways, whereas GPX4 serves to suppress ferroptosis in antioxidant metabolism pathways. To further explore whether AMPK regulates BECN1 and other molecules, such as ACSL4 or GPX4, in the context of CAP-induced ferroptosis, we constructed a stable cell line with AMPK knockdown through lentivirus-mediated shRNA infection (**Figure 4A**). The results indicated that the knockdown of AMPK could reverse the promoting effect of CAP on p-BECN1 and ACSL4 expression, while having no significant effect on GPX4 expression (**Figures 4B, C**). Meanwhile, the heightened release of glutamate following AMPK knockdown indicated an upregulation of SLC7A11 activity (**Figure 4D**). Furthermore, the accumulation of MDA, Fe^2+^, and ROS, along with the reduction of GSH following AMPK knockdown, reinforced that ferroptosis induced by CAP is regulated not only through the AMPK/BECN1 pathway but also via the AMPK/ACSL4 pathway (**Figures 4E–G**).

### 3.5 CAP inhibited tumor growth via AMPK *in vivo*

**Figure 5:**
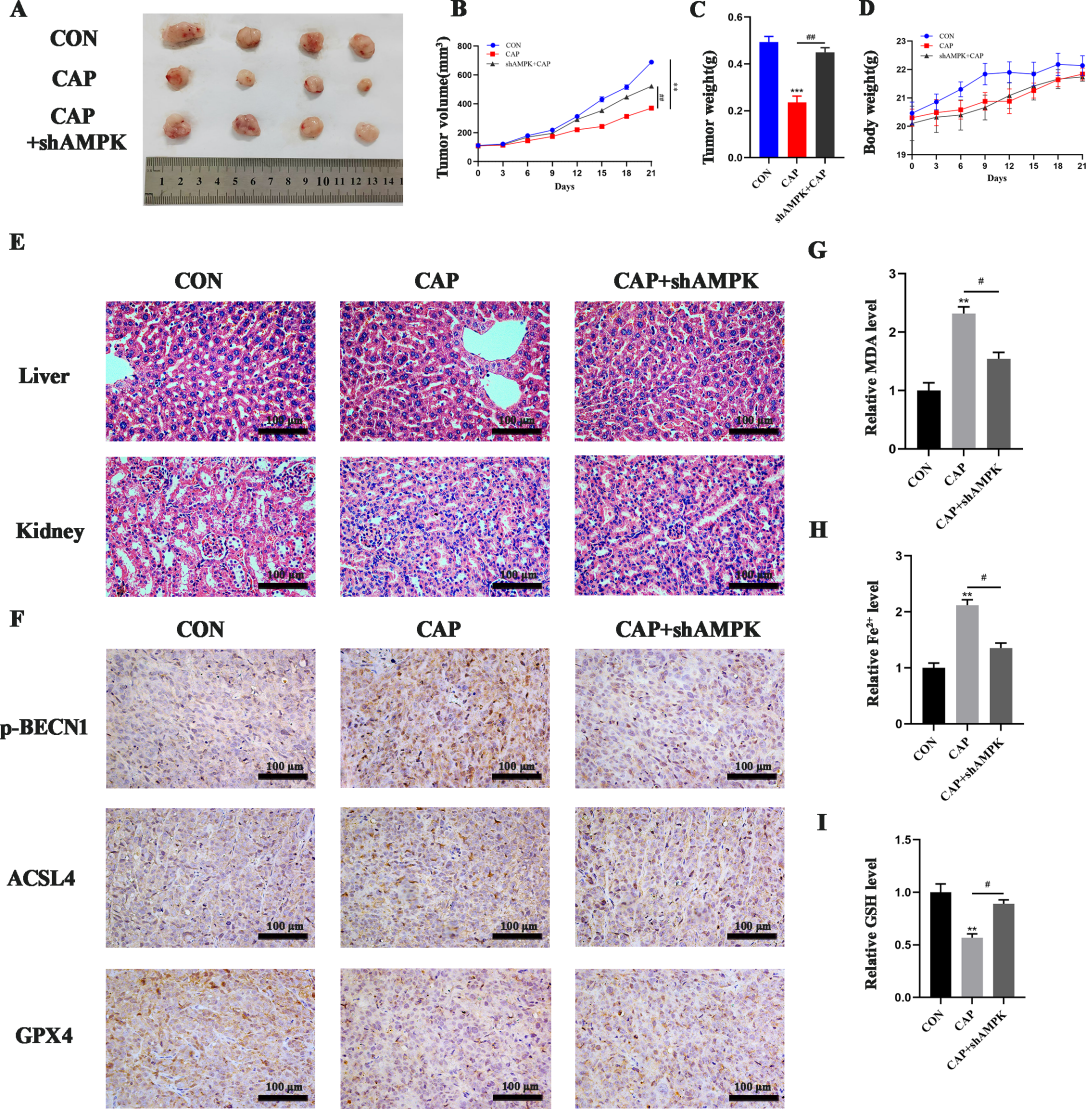
CAP inhibited tumor growth via AMPK *in vivo*. **(A)** Images of the tumors excised following CAP treatment. The volume of tumors **(B)** and the weights of nude mice **(D)** were monitored throughout the CAP treatment. Tumor weight **(C)** was assessed after CAP treatment. **(E)** Histopathological alterations in the liver and kidneys of nude mice following CAP treatment were examined using HE staining. **(F)** immunohistochemical staining for p-BECN1, ACSL4 and GPX4 were conducted to evaluate ferroptosis within the tumor. **(G–I)** MDA **(G)**, Fe^2+^ **(H)** and GSH **(I)** level of tumor tissues were measured after CAP treatment, with or without AMPK knockdown. ** *P* < 0.01, *** *P* < 0.001 vs. CON; ^#^ *P* < 0.05, ^##^ *P* < 0.01 vs. CAP. CON, control; CAP, capsaicin.

*In vitro* experiments have demonstrated that CAP can induce ferroptosis in TSCC cells through the activation of AMPK. We further conducted investigations to determine whether CAP has similar effects *in vivo*. Compared to the control group, CAP significantly reduced tumor volume and weight. Additionally, the tumor volume and weight in the CAP + shAMPK group were notably larger than those in the CAP group, indicating that CAP induced ferroptosis *in vivo* via AMPK (**Figures 5A–C**). There was no significant difference in body weight between the control and treatment groups (**Figure 5D**). No pathological morphological or structural abnormalities were observed in the liver and kidney tissues by HE staining (**Figure 5E**). These findings indicated that CAP has no obviously toxicity to the liver and kidneys of mice. The immunohistochemistry (IHC) results demonstrated that the expression of p-BECN1 and ACSL4 were significantly elevated in the CAP group, while markedly reduced in the group inoculated with shAMPK cells. Nevertheless, the level of GPX4 showed a significant decrease in the CAP group, while no notable recovery was observed in the group inoculated with shAMPK cells (**Figure 5F**). Additionally, elevated levels of MDA and Fe^2+^, along with reduced levels of GSH, were observed in the CAP group, however, these effects were completely reversed in AMPK knockdown group (**Figures 5G–I**), indicating that CAP significantly promotes ferroptosis through AMPK *in vivo*.

## 4 Discussion

In this study, we elucidated that CAP exerts its effects by activating two distinct pathways of AMPK. Firstly, the activated AMPK phosphorylates BECN1 at Ser90, which enhances the binding affinity of BECN1 to SLC7A11 and subsequently inhibits the activity of SLC7A11. Secondly, the activated AMPK upregulates the expression of ACSL4, consequently elevating the level of MDA, which suggests the occurrence of LPO. Collectively, CAP induces ferroptosis by suppression of SLC7A11 activity and upregulation of ACSL4 mediated by AMPK in TSCC cells.

Drawing upon prior research findings (10, 30), we selected the moderate concentration of CAP (150 μM) and ferrostatin-1 (1 μM) for subsequent experiments. The CCK-8 assay showed that CAP significantly inhibited the viability of HN6 and CAL27 cells, while Fer-1, the inhibitor of ferroptosis, could reverse this effect, indicating that the cytotoxicity of CAP in HN6 and CAL27 cells was related to the ferroptosis pathway. Simultaneously, electron microscopy observations revealed the typical damage characteristics of ferroptosis in mitochondria, such as the dwindle in size, rupture of membrane and fragmentation of cristae (35). Additionally, CAP significantly increased the levels of ferroptosis biomarkers such as MDA, Fe²^+^, and ROS, while decreasing GSH levels. Notably, all effects induced by CAP were reversed following pre-treatment with Fer-1. The process of ferroptosis encompasses various regulatory mechanisms, including amino acid metabolism, lipid metabolism, and iron metabolism (36). Key regulatory molecules involved GPX4, SLC7A11 and ACSL4. We found that CAP significantly downregulated the expression of GPX4 and upregulated the expression of ACSL4, and the effects could be reversed by Fer-1. Interestingly, CAP did not alter the expression of SLC7A11. These results demonstrated that CAP could induce ferroptosis in TSCC cells.

In the present study, the expression of SLC7A11 remained stable after CAP treatment, whether its activity was affected remained unclear? It’s reported that the AMPK/BECN1 pathway was responsible for the absence of changes in SLC7A11 (25). Mechanistically, activated AMPK mediates the phosphorylation of BECN1, promoting the formation of the BECN1-SLC7A11 complex and inhibiting SLC7A11 activity to ultimately induce ferroptosis. Our previous research has confirmed that CAP activates AMPK in TSCC (16). Here, we furtherly revealed that CAP significantly enhanced the AMPK-mediated phosphorylation of BECN1, promoted the binding of BECN1 to SLC7A11, and concurrently suppressed the activity of SLC7A11 in HN6 and CAL27 cells. The knockdown of AMPK reversed the effects of CAP on BECN1 phosphorylation and the inhibition of SLC7A11 activity. These findings indicated that the activity of SLC7A11 was suppressed by AMPK/BECN1 pathway, which contributed to the ferroptosis induced by CAP in TSCC.

GPX4 was the signal downstream of SLC7A11 in the AMPK/BECN1/SLC7A11 pathway. We discovered that CAP inhibited the protein expression of GPX4, meanwhile, regulated the activity of GPX4 through AMPK, which demonstrated by the level of GSH after activation or knockdown of AMPK. However, knockdown of AMPK does not reverse the decrease of GPX4 expression, further studies are necessary to elucidate its underlying mechanisms.

AMPK is integral to the regulation of energy metabolism. Its activation typically inhibits fatty acid synthesis while simultaneously promoting β-oxidation, thereby reducing fat storage and ensuring an adequate supply of cellular energy (37). Furthermore, ACSL4 significantly influences lipid metabolism (22). Zhao et al. discovered that the activation of AMPK can reduce the expression of stearoyl-CoA desaturase-1 (SCD1) and work synergistically with ACSL4, thus increasing the sensitivity of hepatocellular carcinoma to ferroptosis (38). In our study, to figure out the relationship between AMPK and ACSL4, AMPK was stably knocked down *in vitro* and the upregulation of ACSL4 induced by CAP was reversed. Furthermore, upregulation of MDA, Fe^2+^, ROS and downregulation of GSH induced by CAP were also reversed. These findings indicated that CAP-induced ferroptosis in TSCC occurs, at least in part, through the upregulation of ACSL4 mediated by AMPK activation, ultimately leading to the inhibition of TSCC growth. Additionally, we demonstrated that the ferroptosis-promoting effect of CAP *in vivo* is mediated through AMPK, consistent with the results *in vitro*.

**Figure 6:**
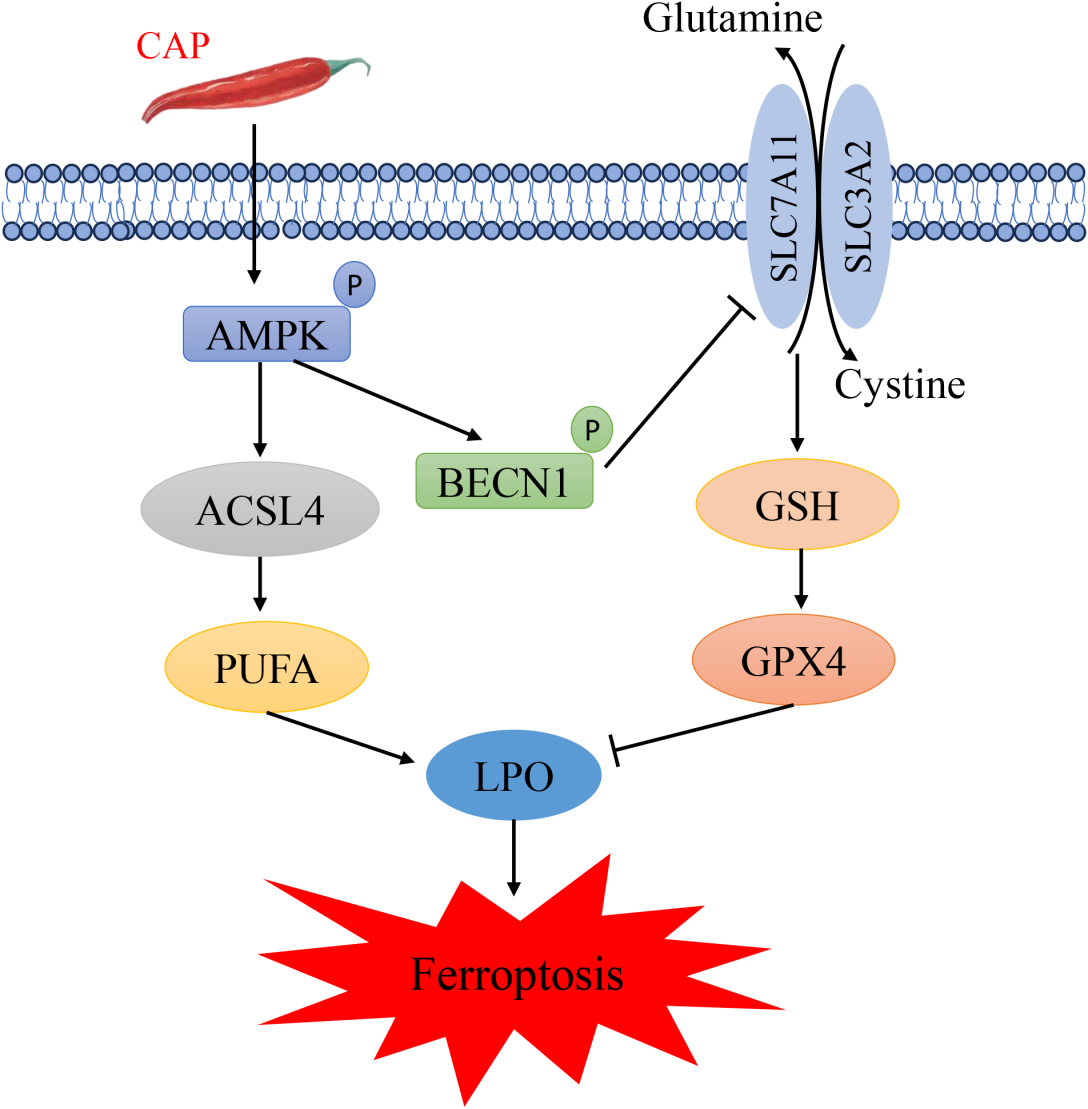
Schematic diagram illustrating the potential mechanism of capsaicin in inhibiting tongue squamous cell carcinoma.

In summary, our findings indicated that CAP induced ferroptosis in TSCC through AMPK/BECN1/SLC7A11 and AMPK/ACSL4 pathways (**Figure 6**). These results suggested that CAP may serve as a promising anti-cancer agent, offering a novel therapeutic strategy for clinical applications.

## Data Availability

The original contributions presented in the study are included in the article/**Supplementary Material**. Further inquiries can be directed to the corresponding author.
