# Supplementary figures and images for: Capsaicin induces ferroptosis via suppression of SLC7A11 activity and upregulation of ACSL4 mediated by AMPK in tongue squamous cell carcinoma
Source: Front Oncol. 2025 May 14;15:1532555. doi: 10.3389/fonc.2025.1532555 (PMC12116642; doi:10.3389/fonc.2025.1532555)

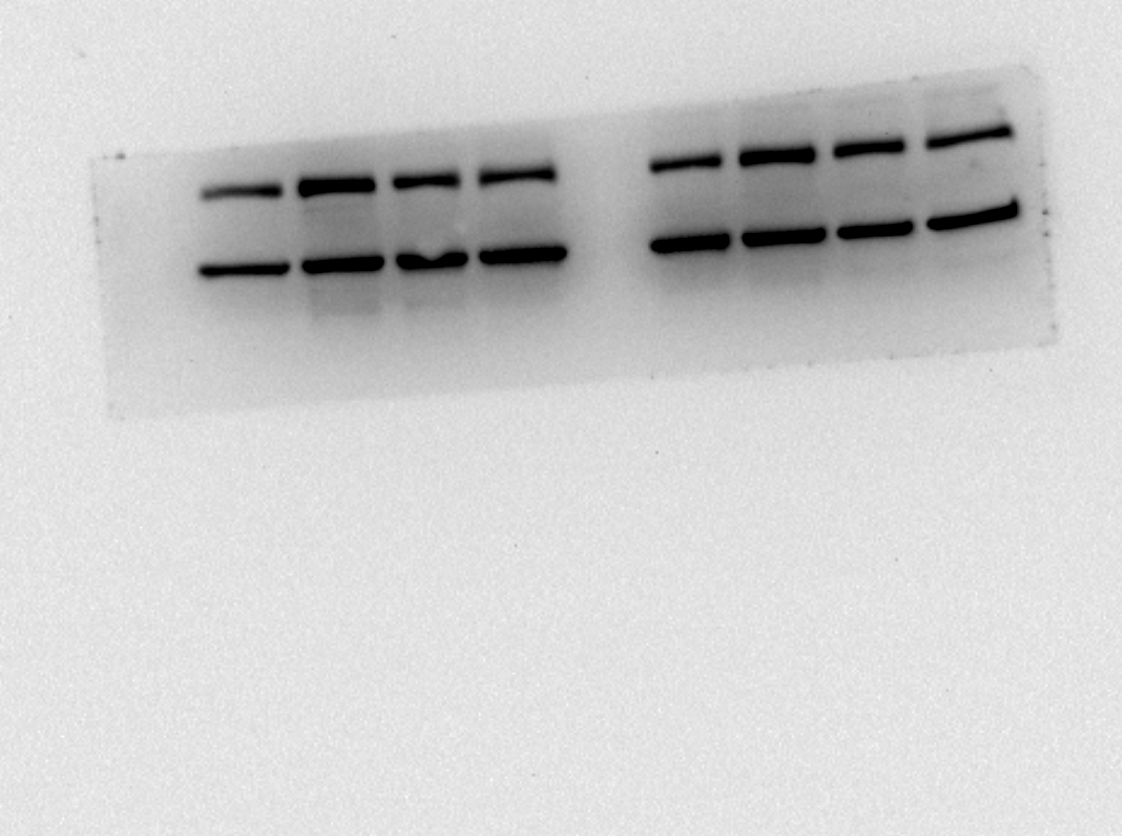

Supplement: Supplementary file 1 [file DataSheet1.zip › Fig2-ACSL4-β-actin.tif]

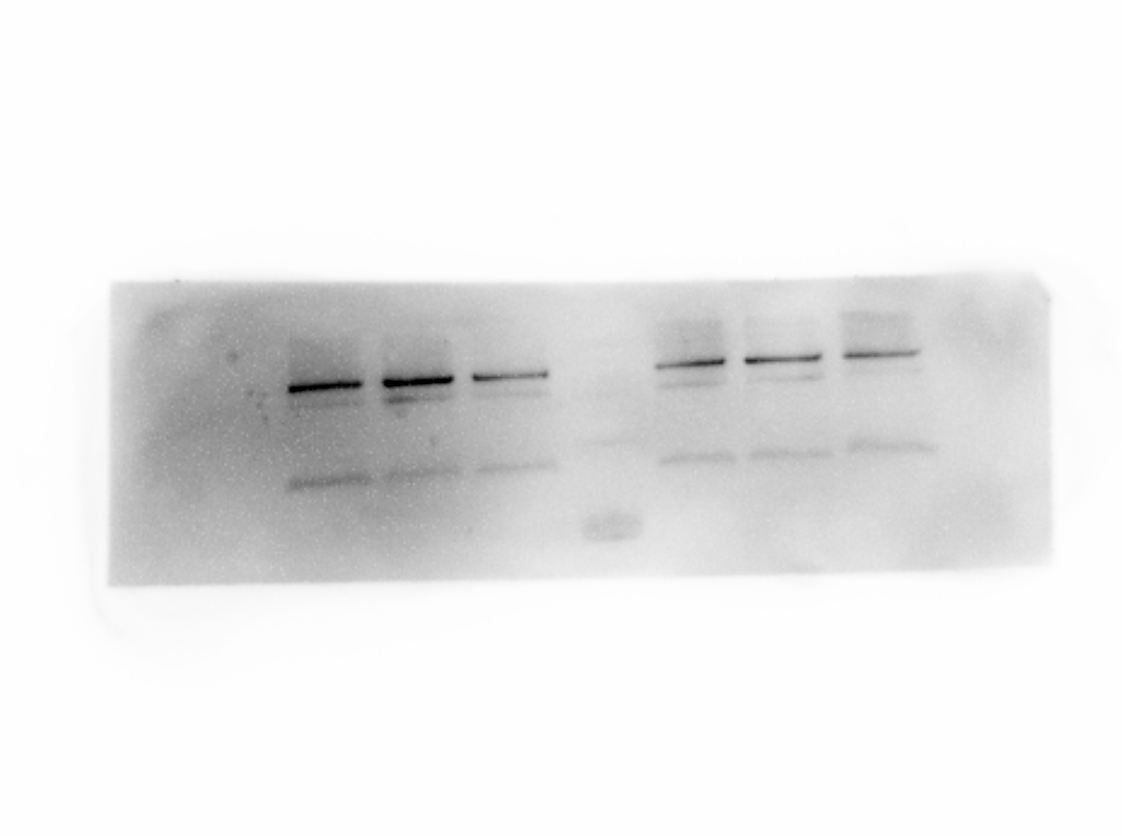

Supplement: Supplementary file 1 [file DataSheet1.zip › Fig2-GPX4-β-actin.tif]

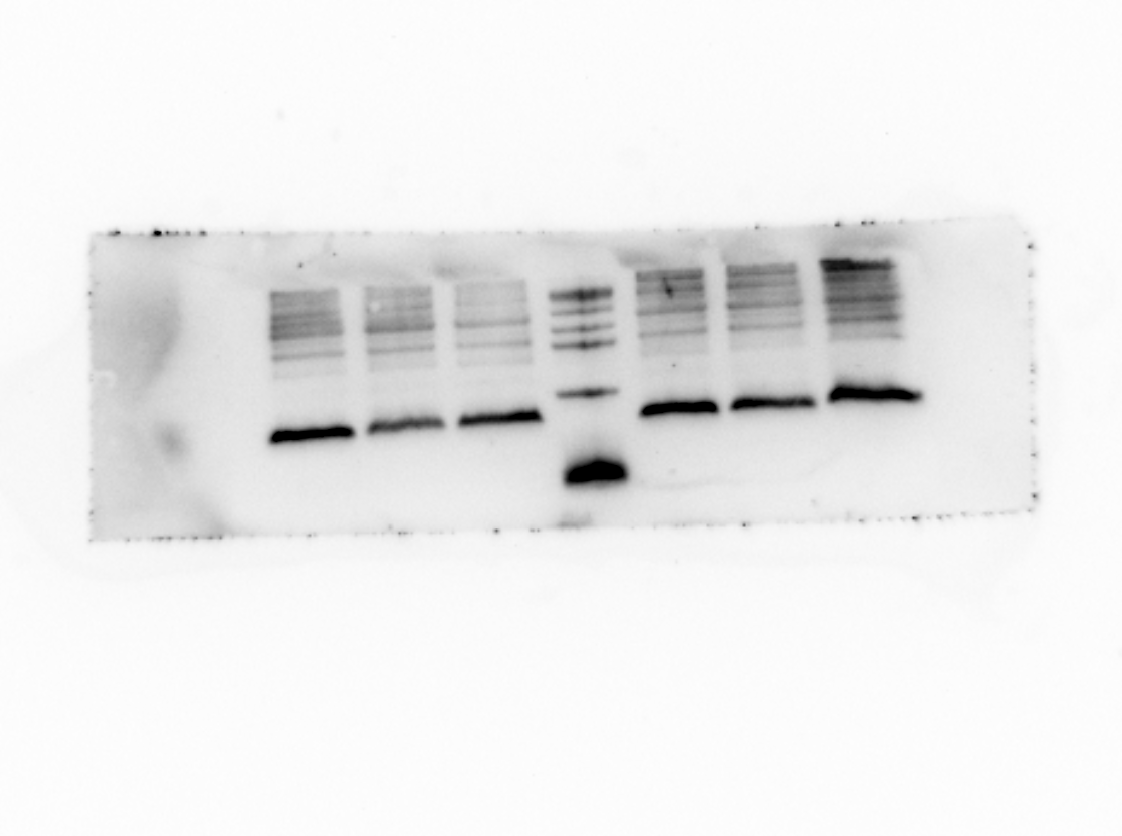

Supplement: Supplementary file 1 [file DataSheet1.zip › Fig2-GPX4.tif]

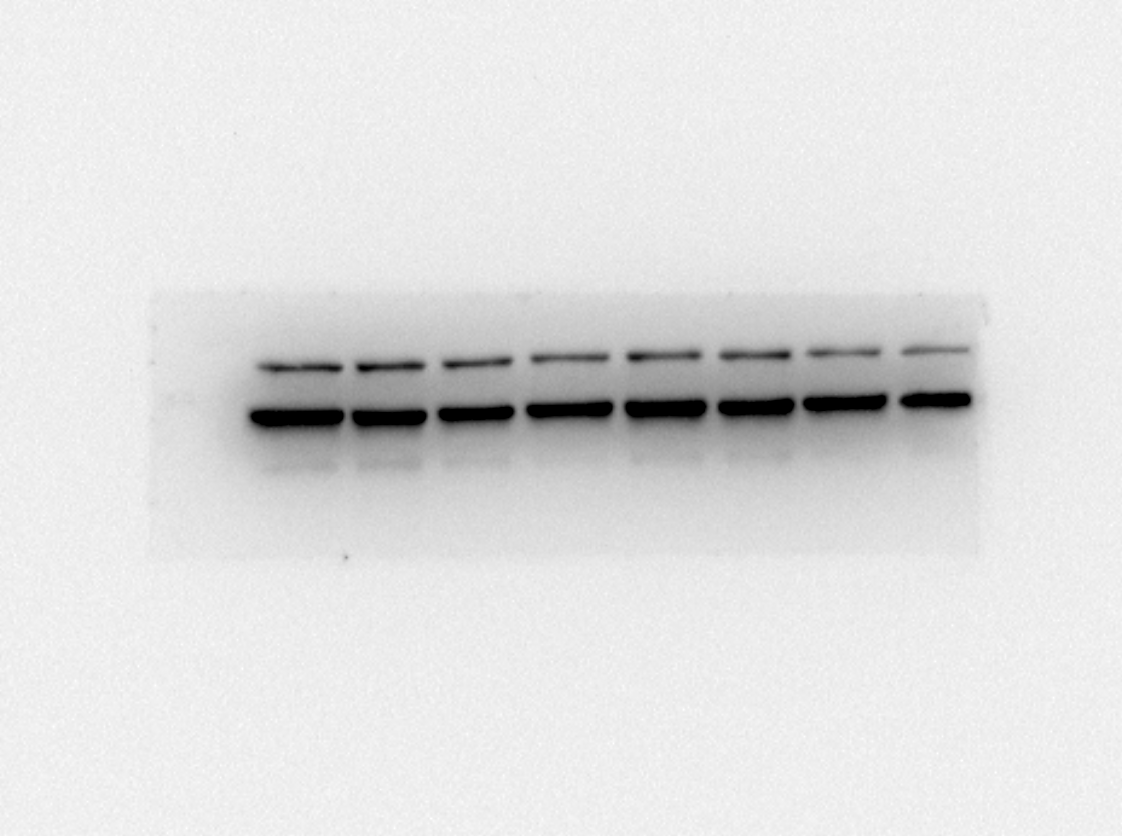

Supplement: Supplementary file 1 [file DataSheet1.zip › Fig2-SLC7A11-β-actin.tif]

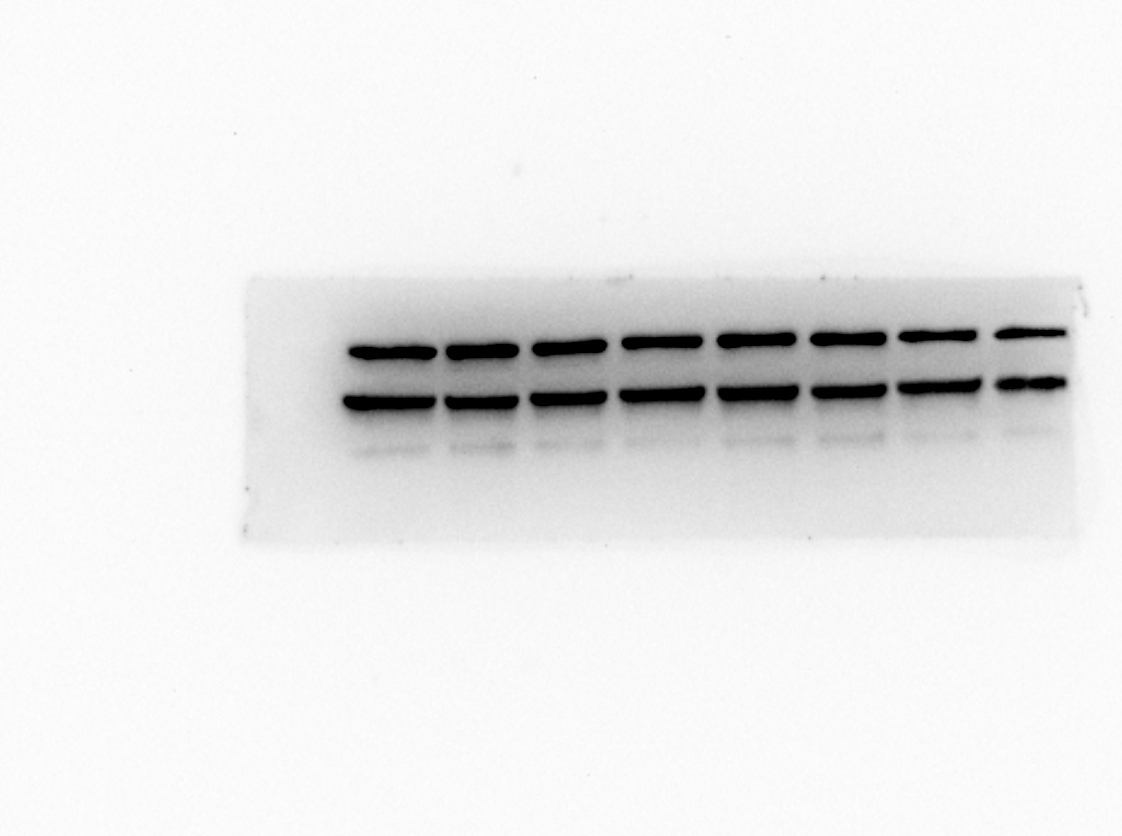

Supplement: Supplementary file 1 [file DataSheet1.zip › Fig2-SLC7A11.tif]

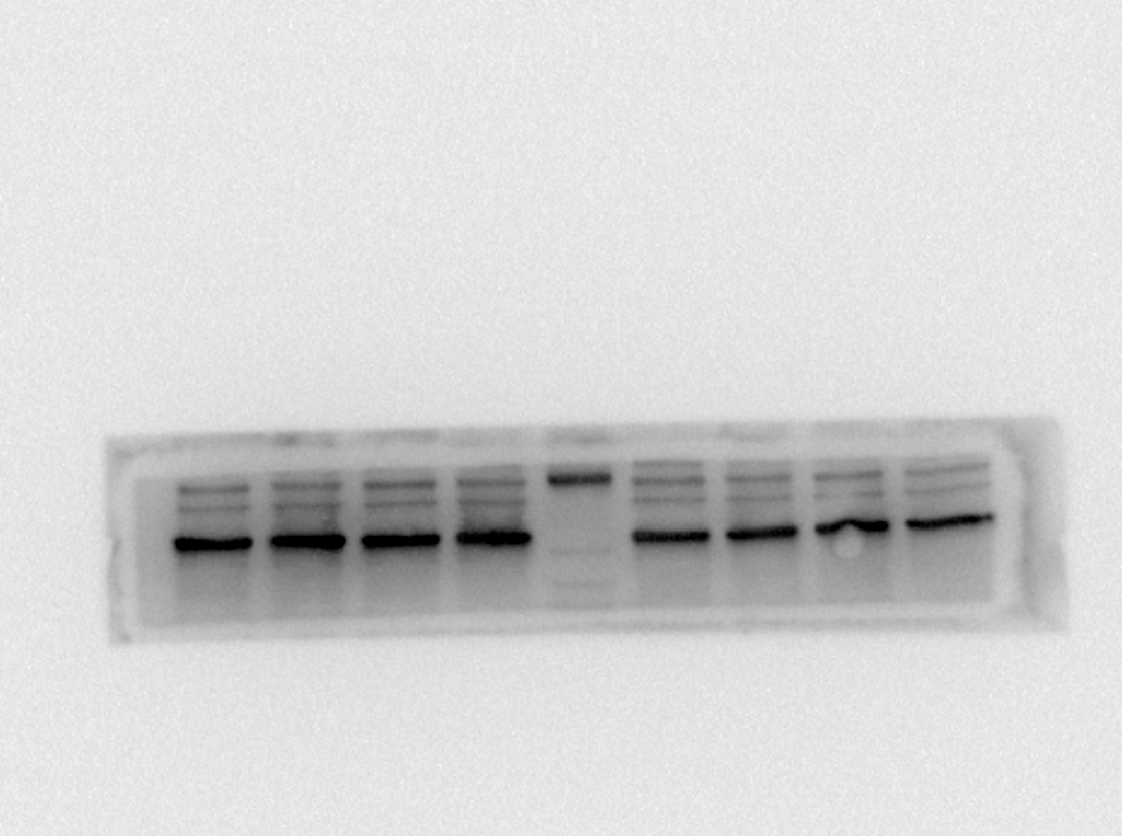

Supplement: Supplementary file 1 [file DataSheet1.zip › Fig3-AMPK-β-actin.tif]

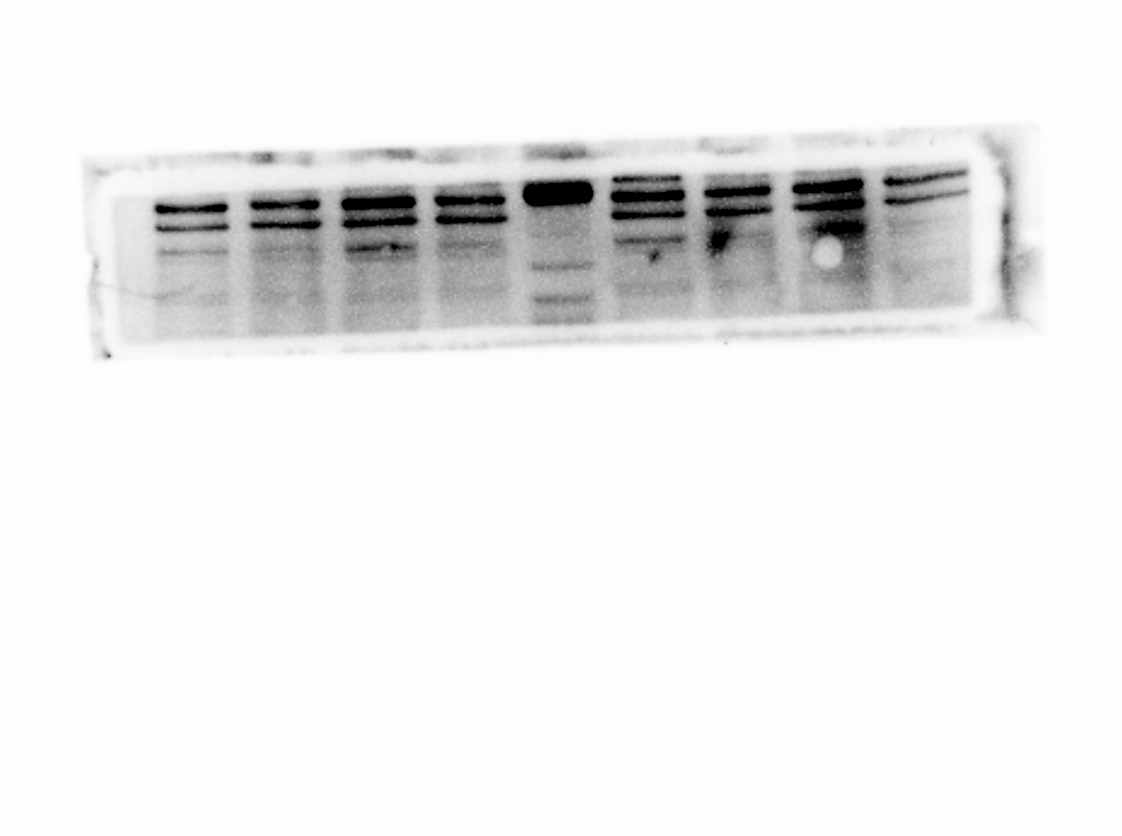

Supplement: Supplementary file 1 [file DataSheet1.zip › Fig3-AMPK.tif]

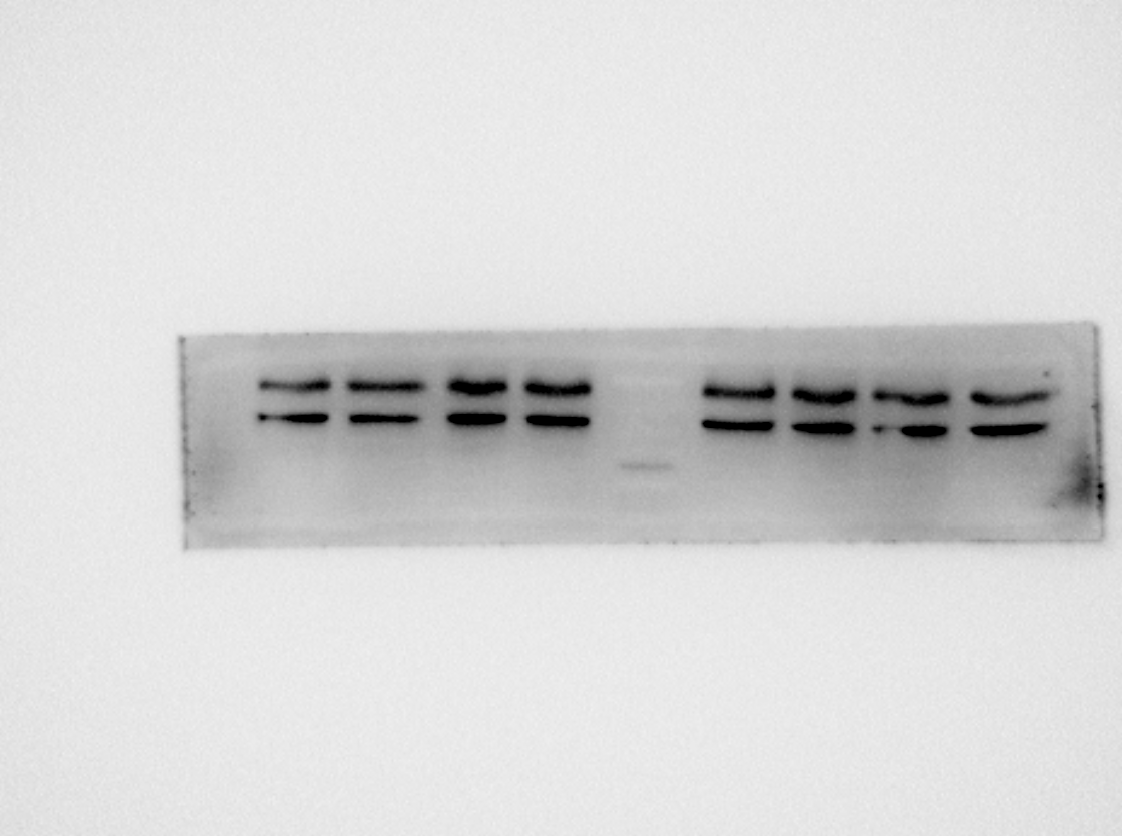

Supplement: Supplementary file 1 [file DataSheet1.zip › Fig3-BECN1-β-actin.tif]

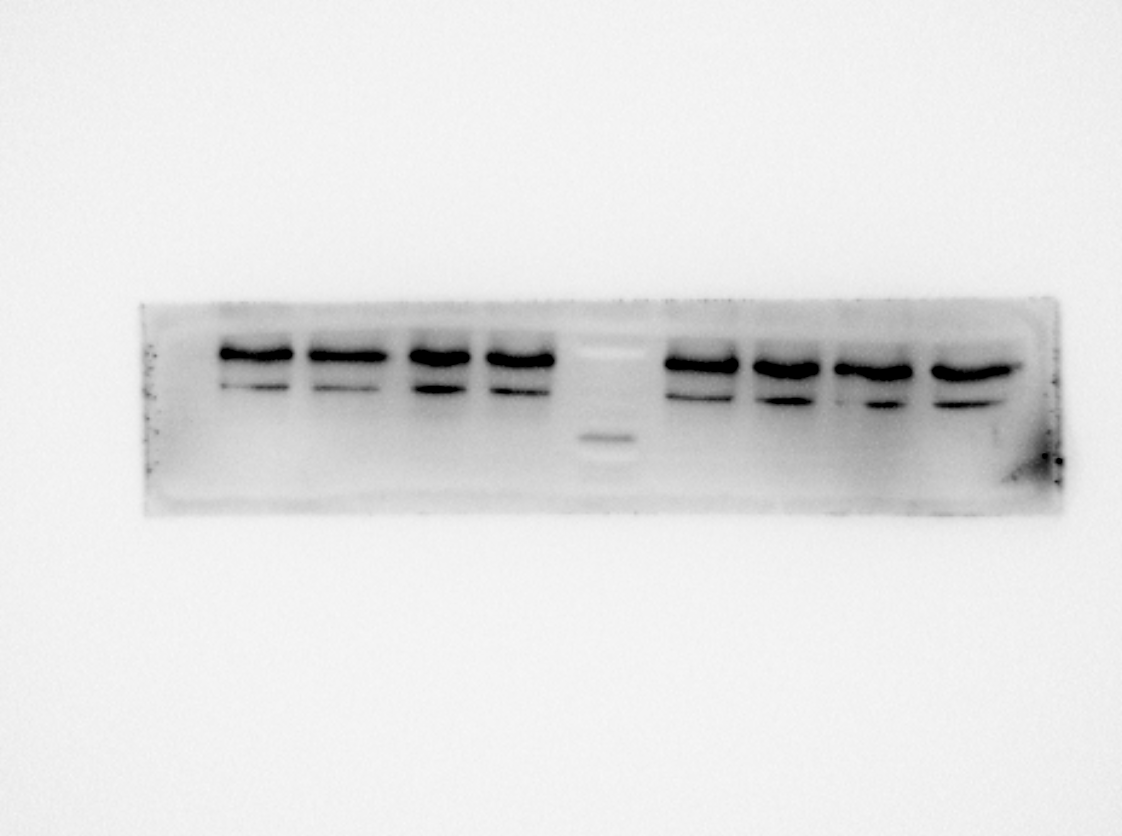

Supplement: Supplementary file 1 [file DataSheet1.zip › Fig3-BECN1.tif]

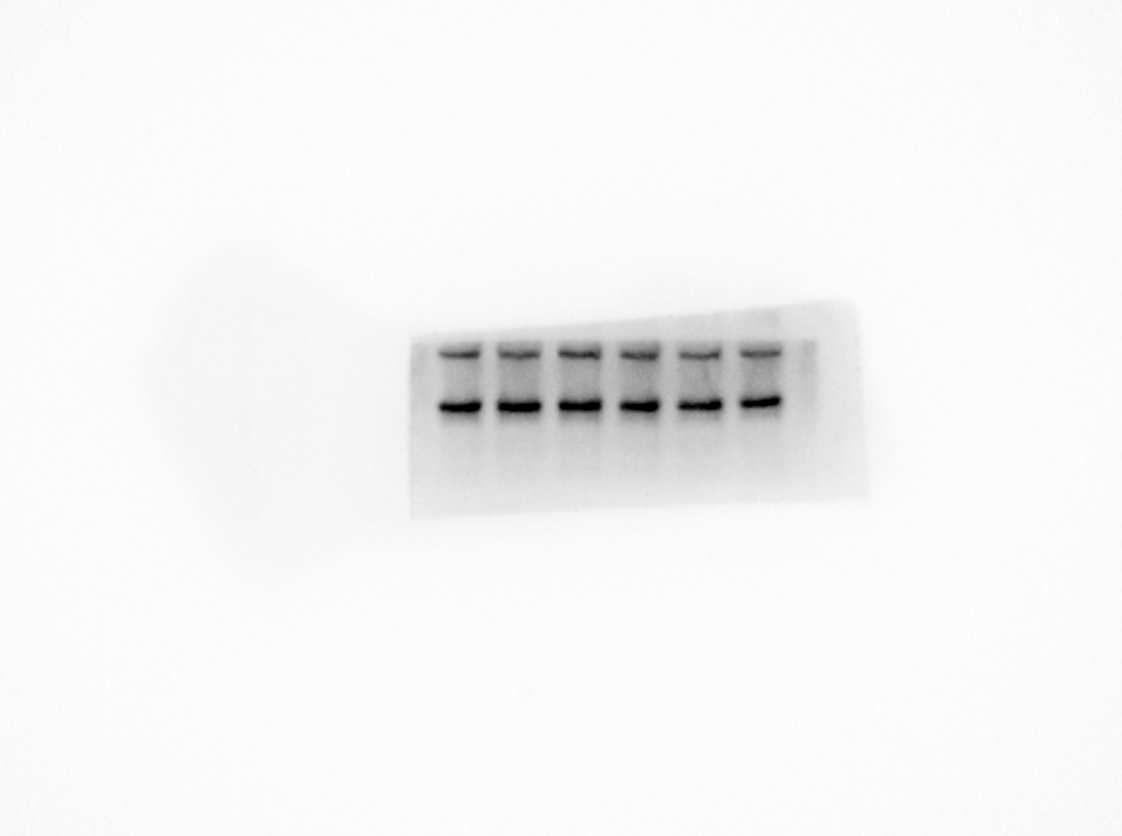

Supplement: Supplementary file 1 [file DataSheet1.zip › Fig3-CAL27Input-BECN1-β-actin.tif]

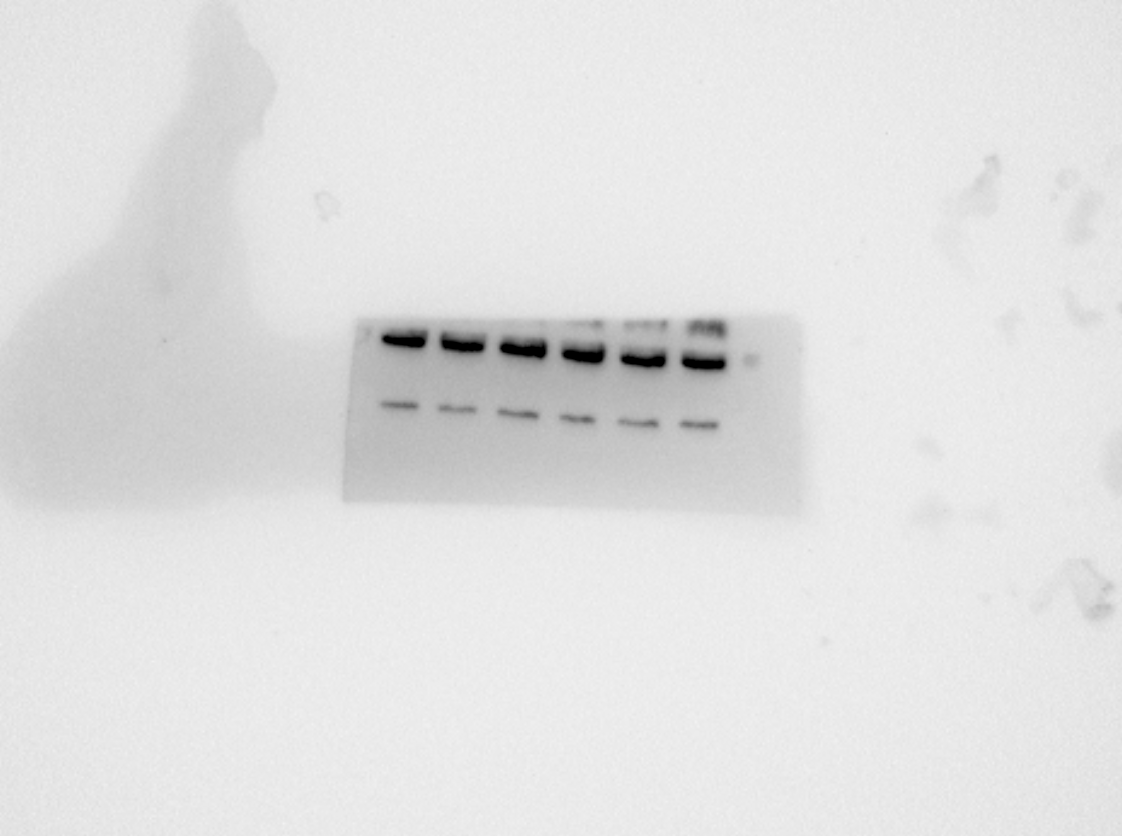

Supplement: Supplementary file 1 [file DataSheet1.zip › Fig3-CAL27Input-BECN1.tif]

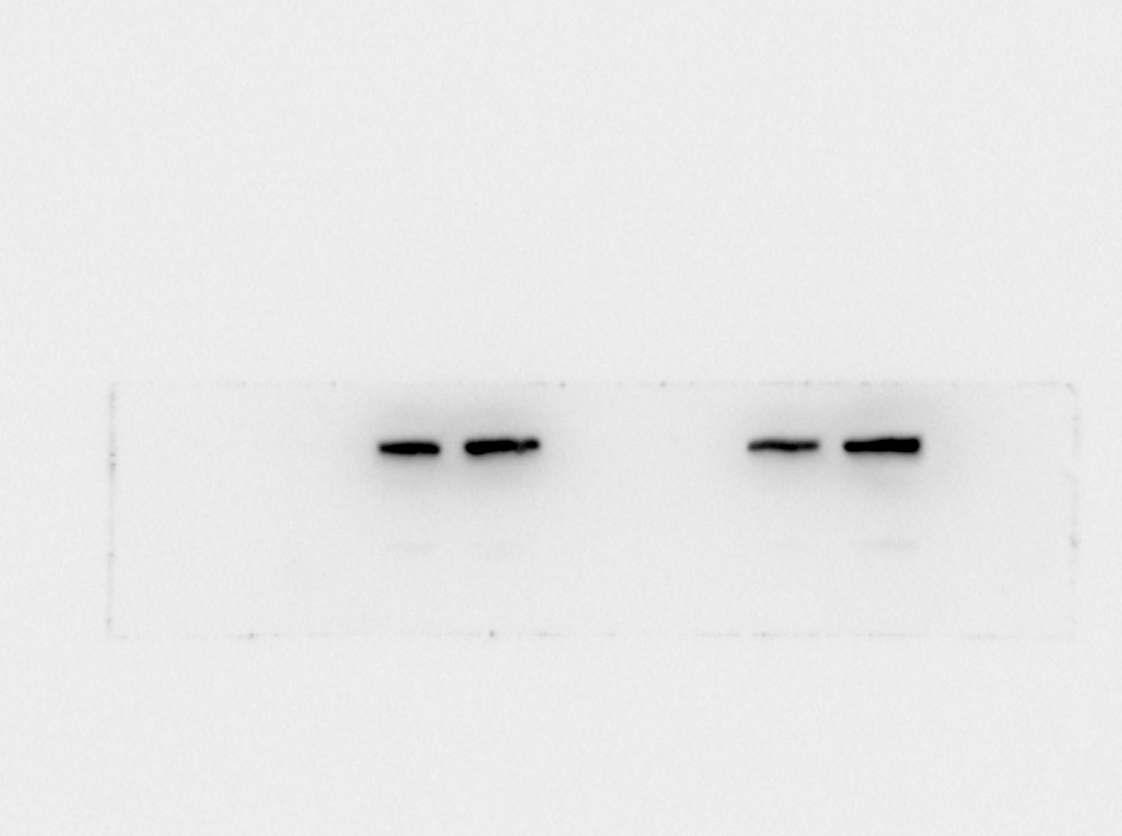

Supplement: Supplementary file 1 [file DataSheet1.zip › Fig3-CAL27IP.tif]

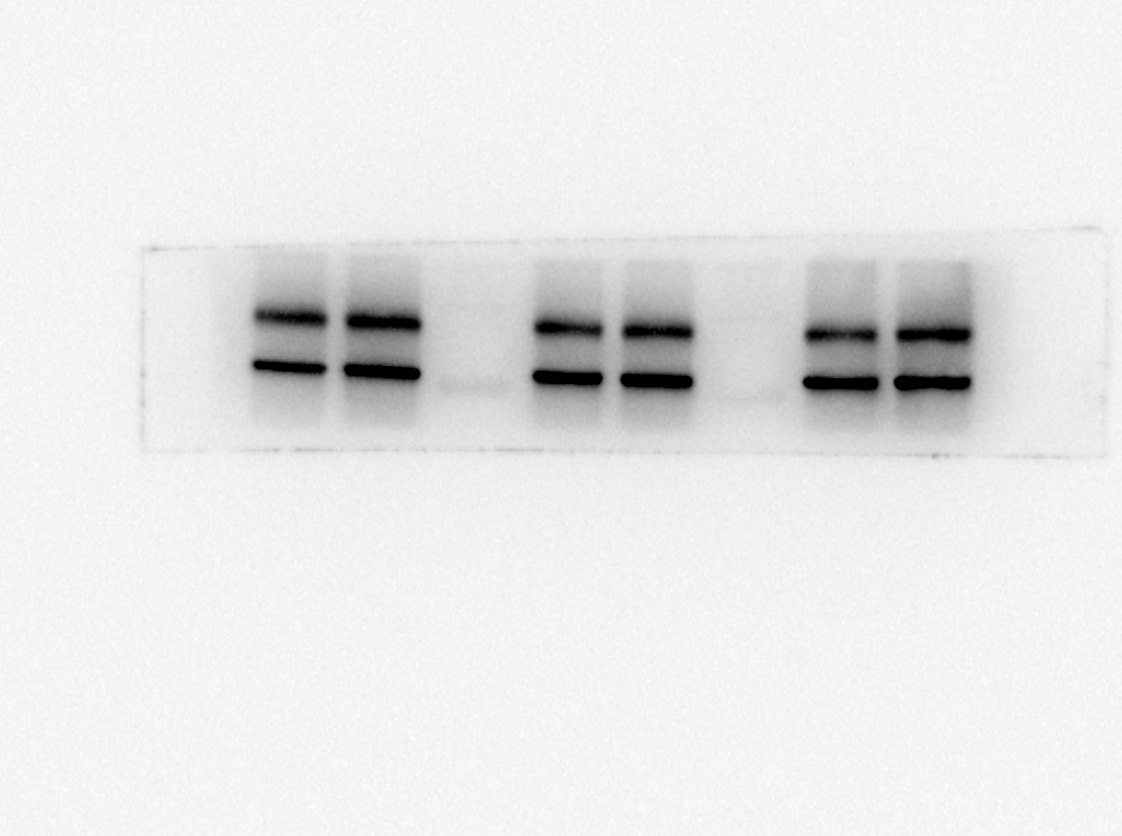

Supplement: Supplementary file 1 [file DataSheet1.zip › Fig3-HN6Input-BECN1-β-actin.tif]

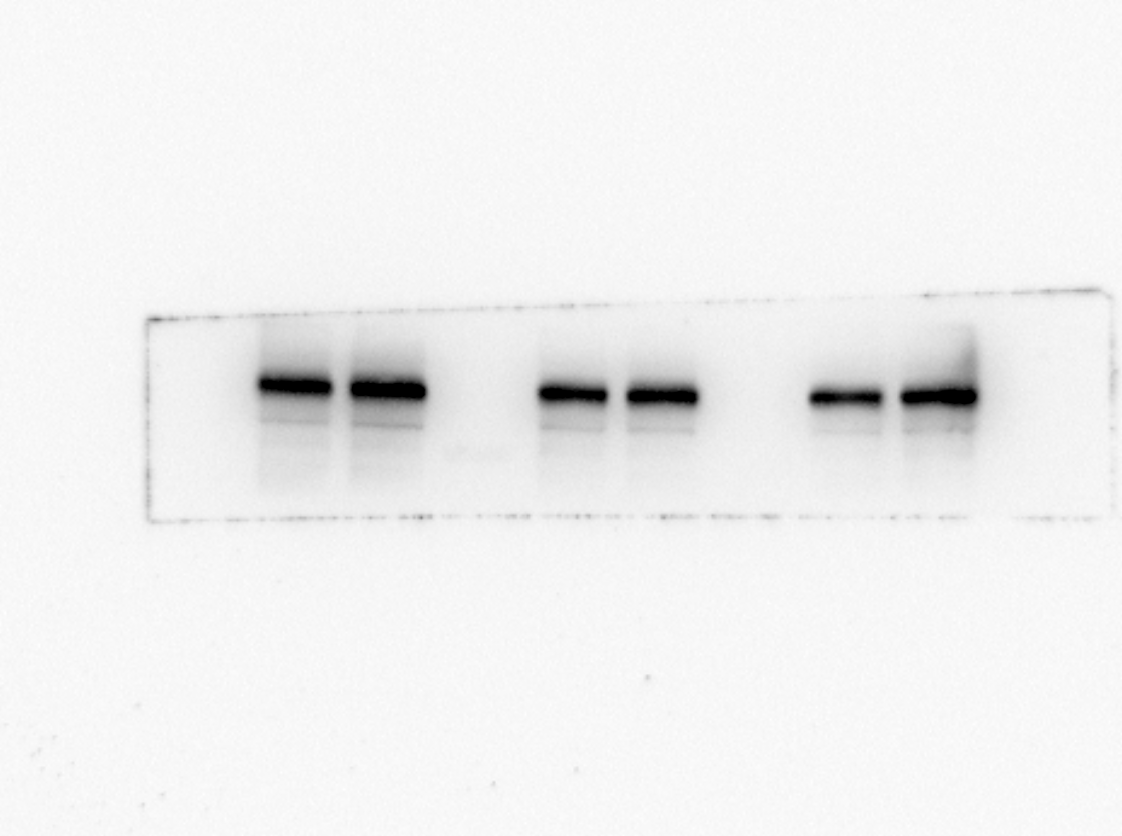

Supplement: Supplementary file 1 [file DataSheet1.zip › Fig3-HN6Input-BECN1.tif]

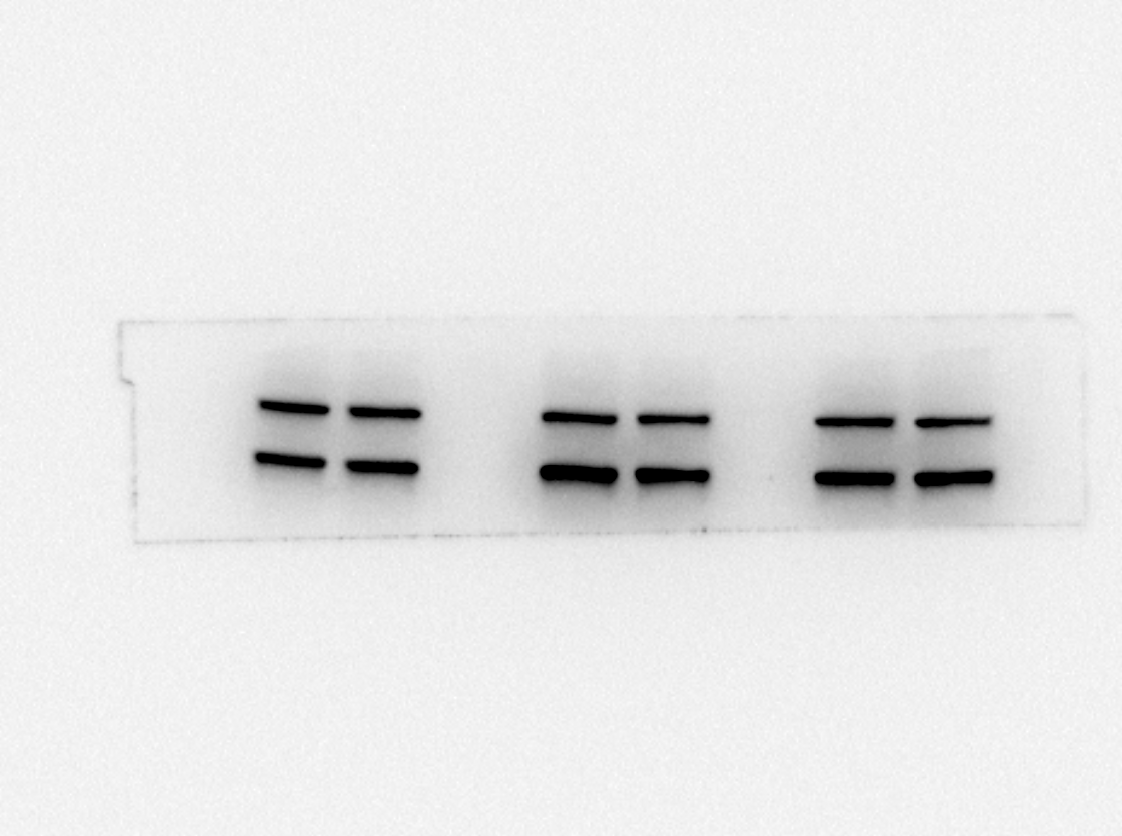

Supplement: Supplementary file 1 [file DataSheet1.zip › Fig3-HN6Input-SLC7A11-β-actin.tif]

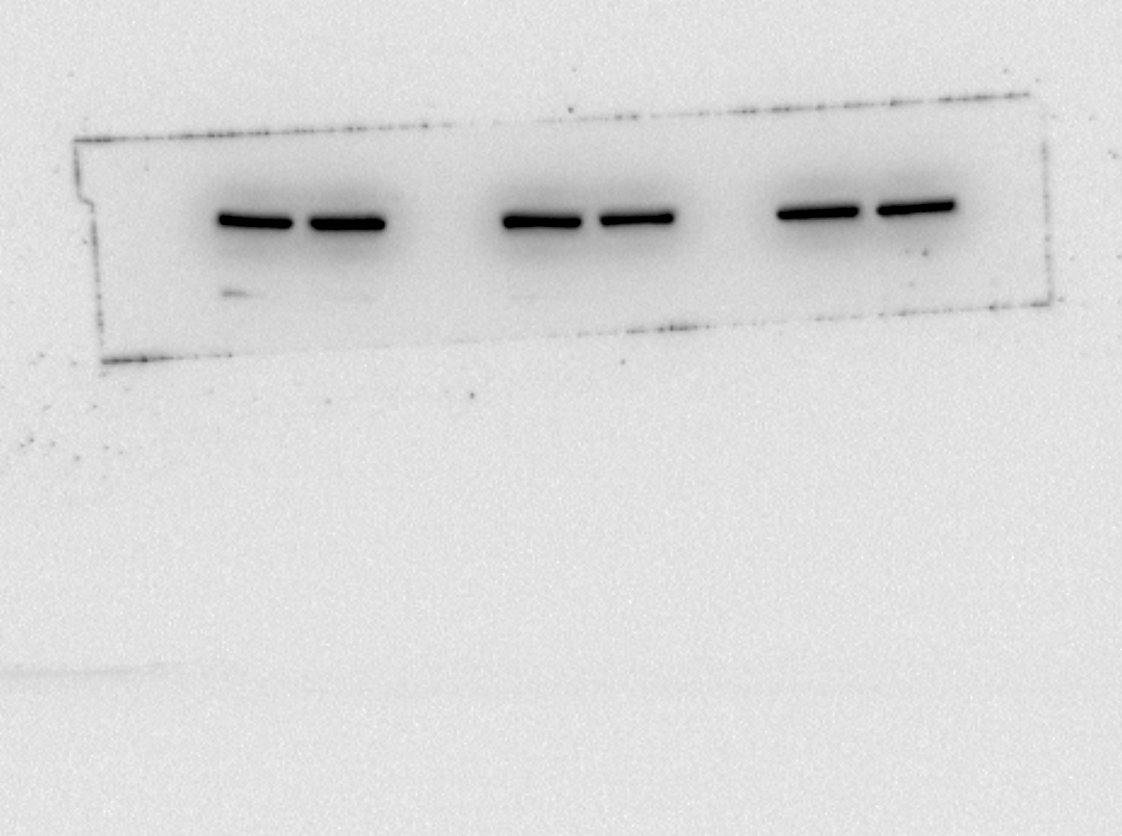

Supplement: Supplementary file 1 [file DataSheet1.zip › Fig3-HN6Input-SLC7A11.tif]

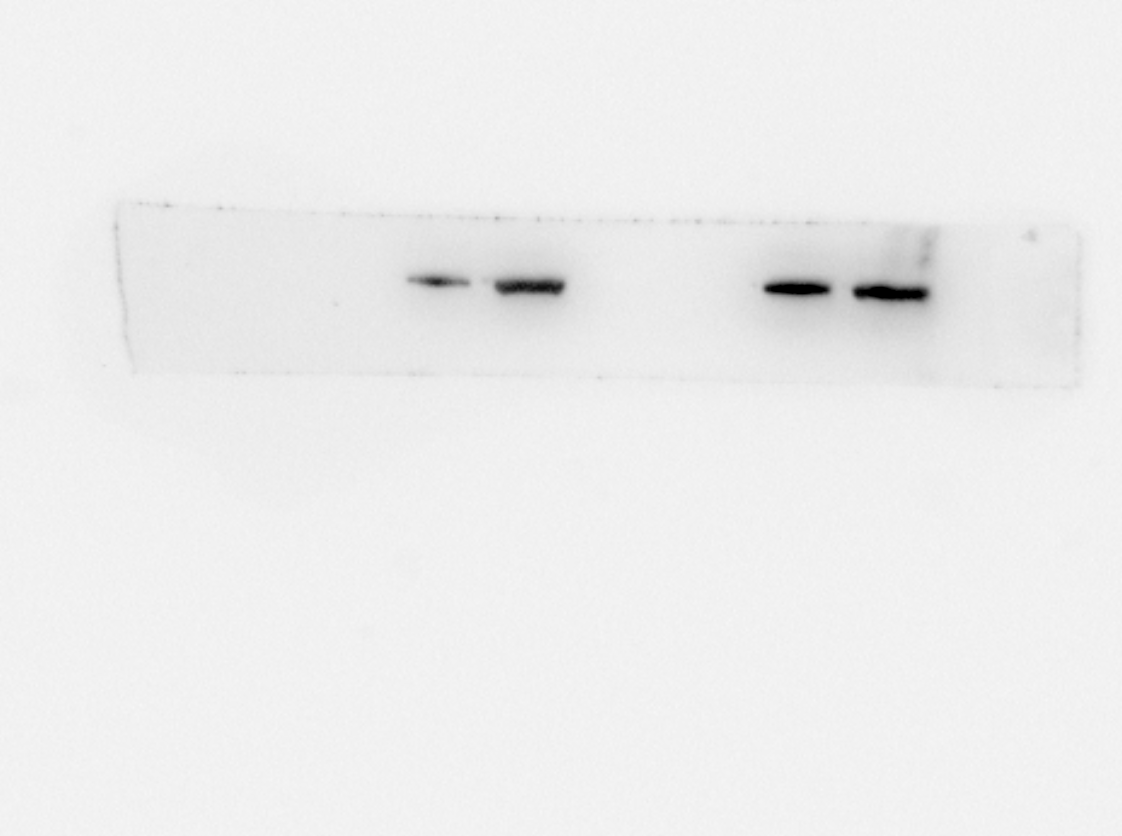

Supplement: Supplementary file 1 [file DataSheet1.zip › Fig3-HN6IP.tif]

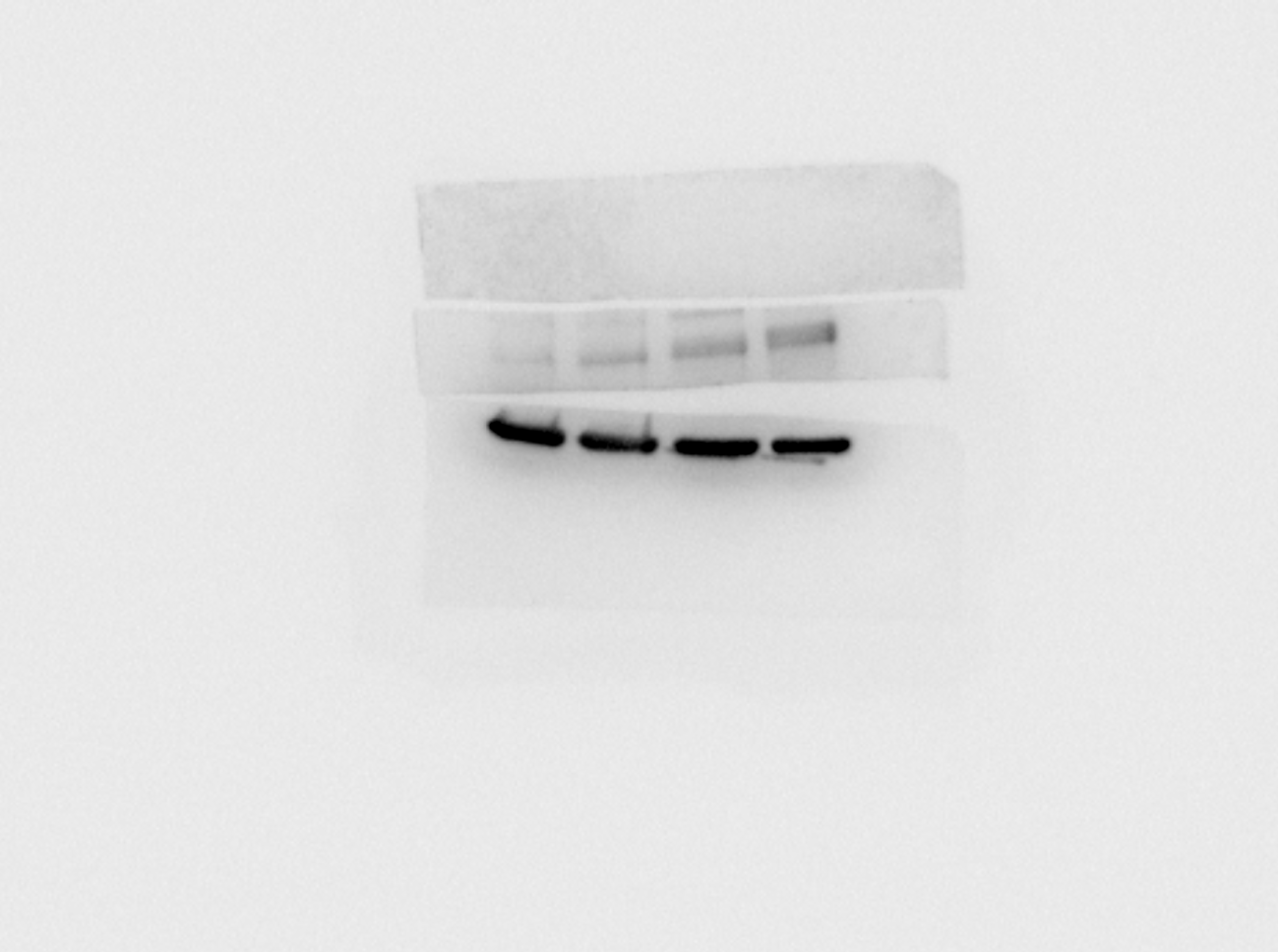

Supplement: Supplementary file 1 [file DataSheet1.zip › Fig3-p-AMPK-β-actin.tif]

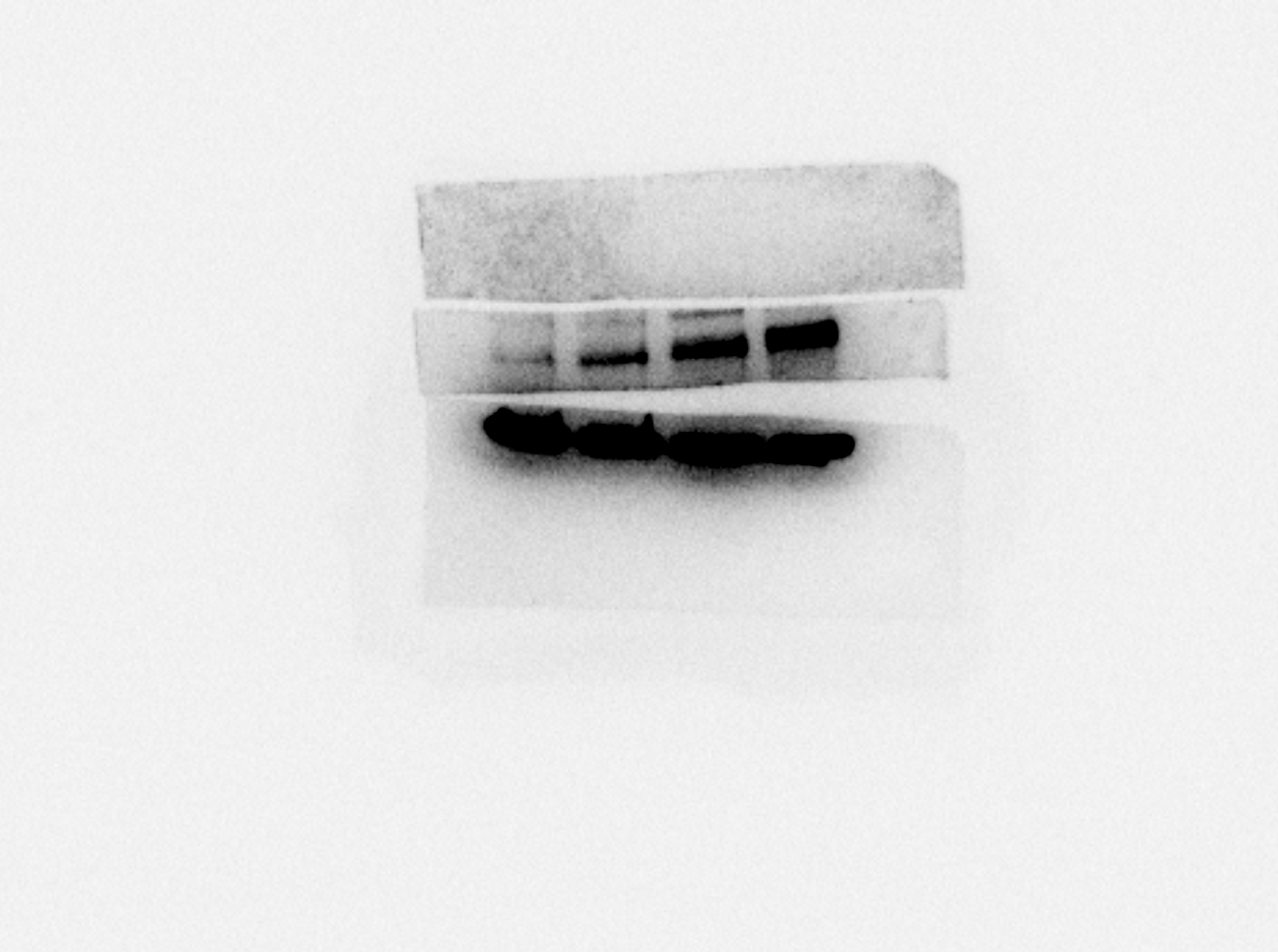

Supplement: Supplementary file 1 [file DataSheet1.zip › Fig3-p-AMPK.tif]

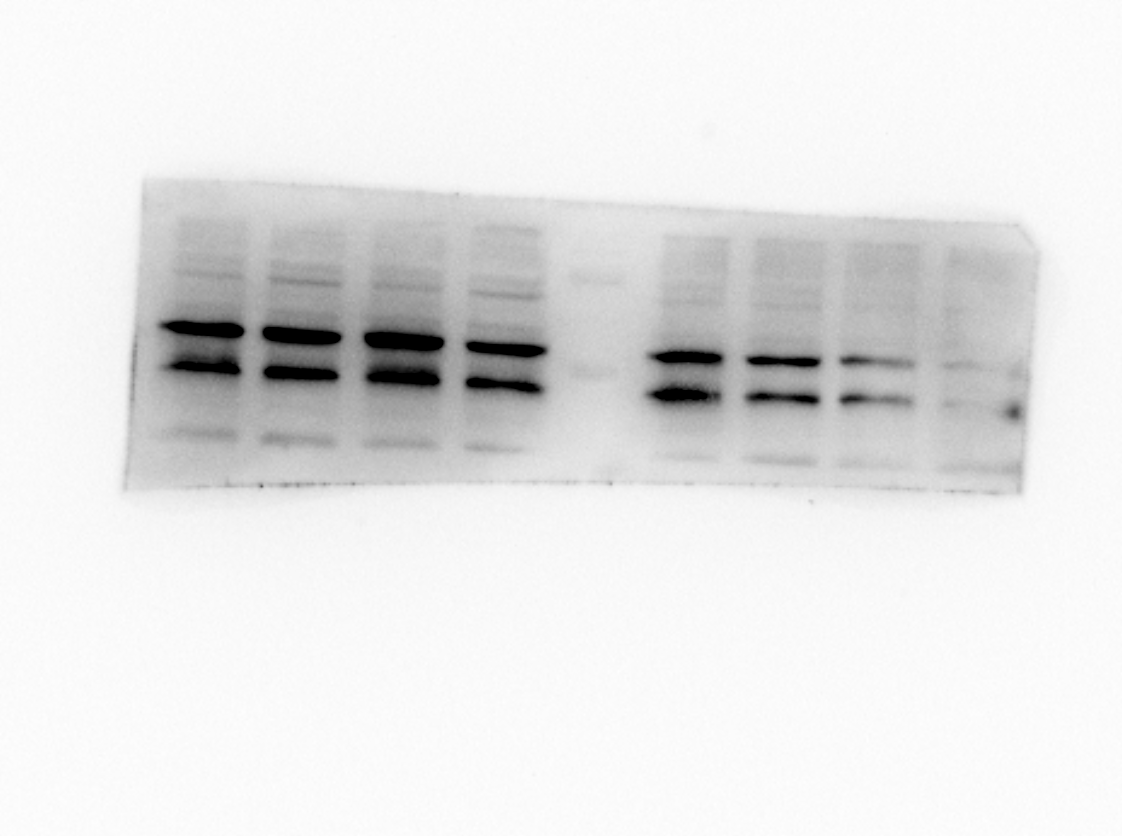

Supplement: Supplementary file 1 [file DataSheet1.zip › Fig3-p-BECN1-β-actin.tif]

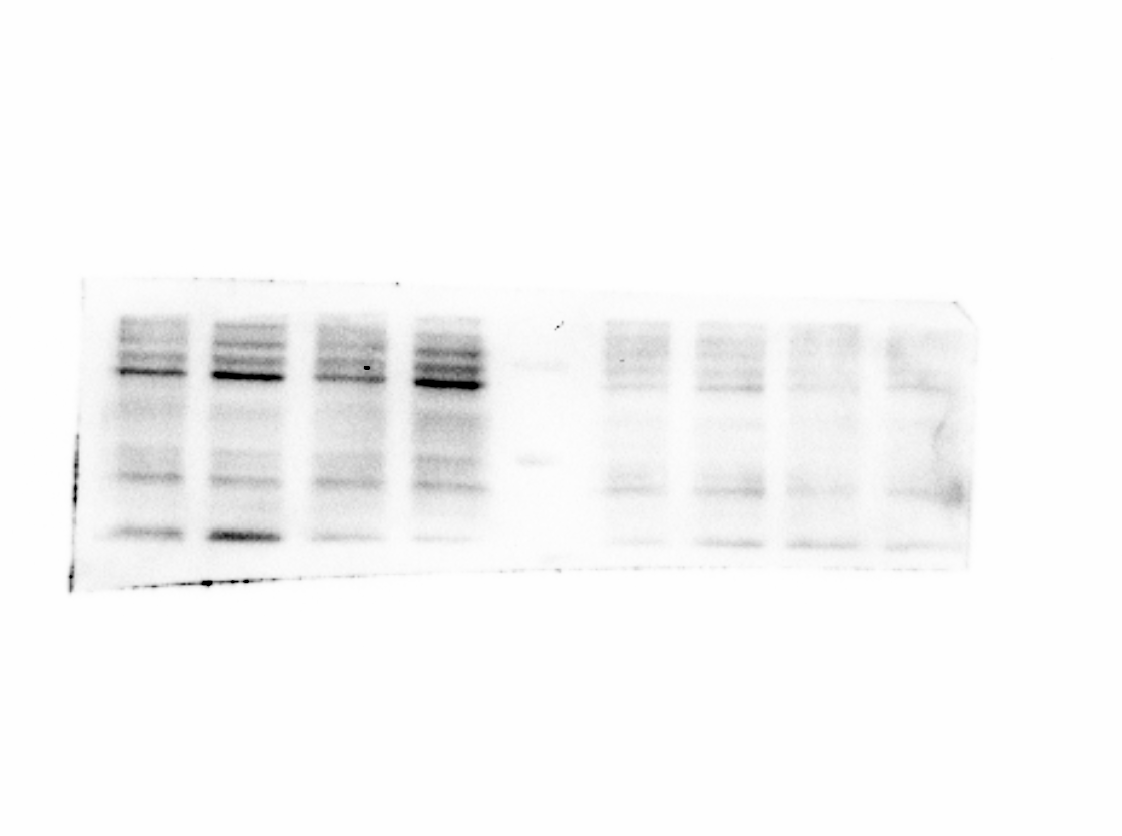

Supplement: Supplementary file 1 [file DataSheet1.zip › Fig3-p-BECN1.tif]

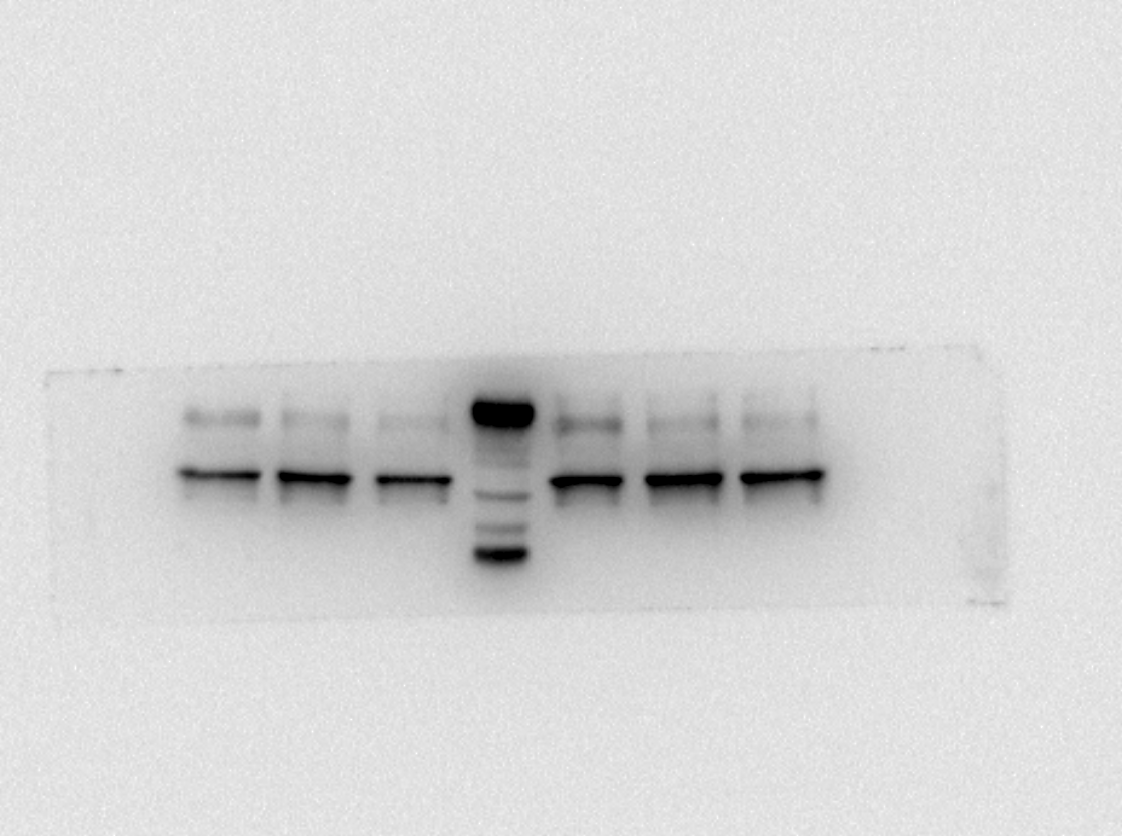

Supplement: Supplementary file 1 [file DataSheet1.zip › Fig4-AMPK-β-actin.tif]

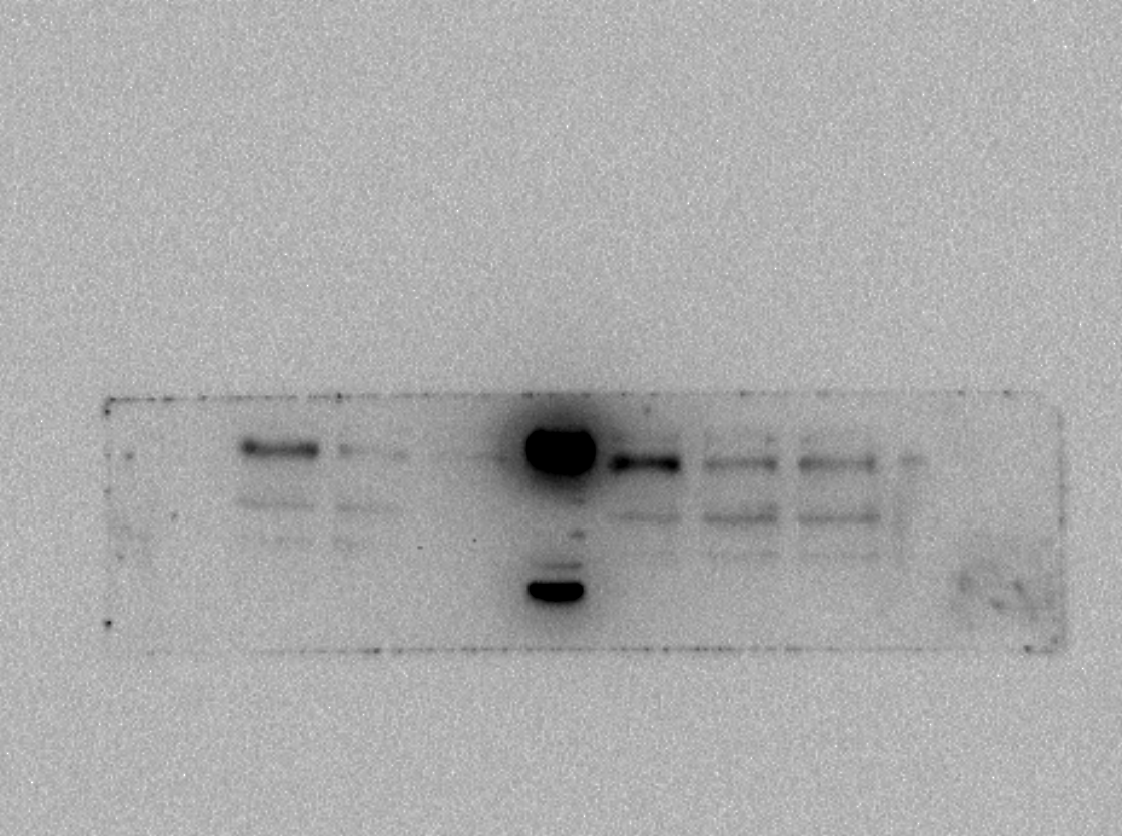

Supplement: Supplementary file 1 [file DataSheet1.zip › Fig4-AMPK.tif]

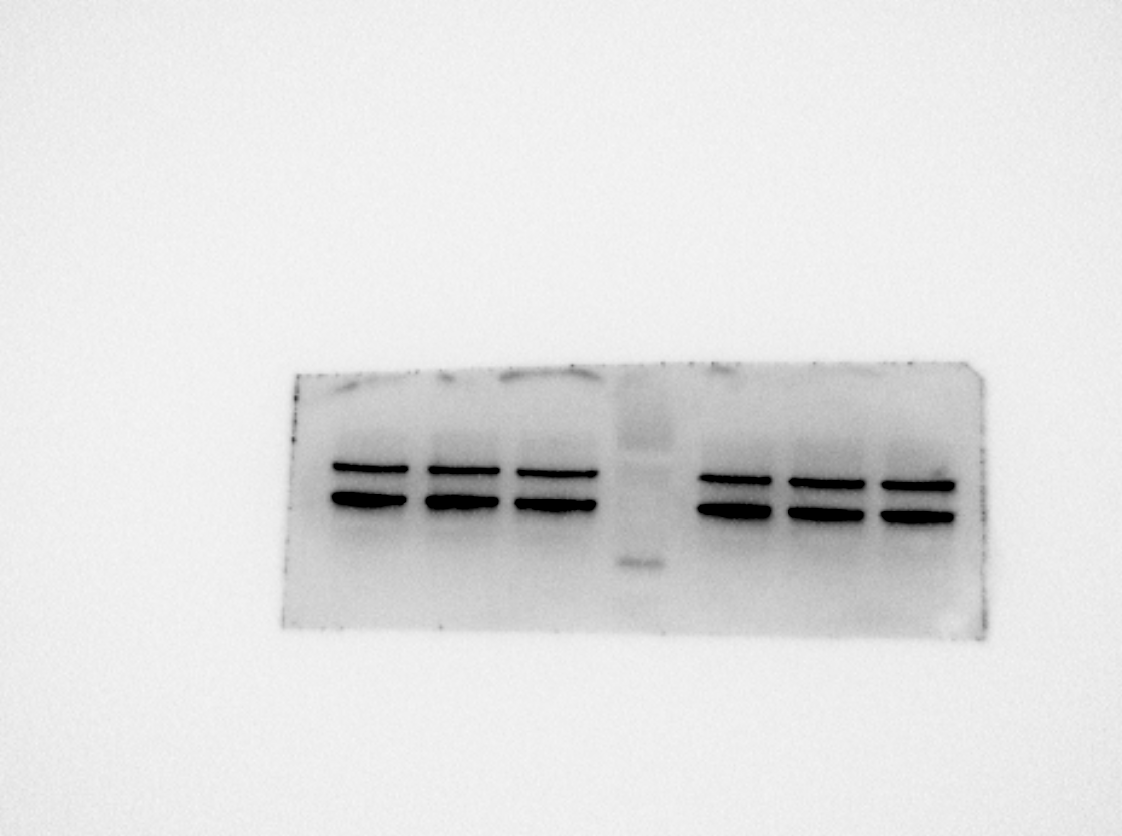

Supplement: Supplementary file 1 [file DataSheet1.zip › Fig4-BECN1-β-actin.tif]

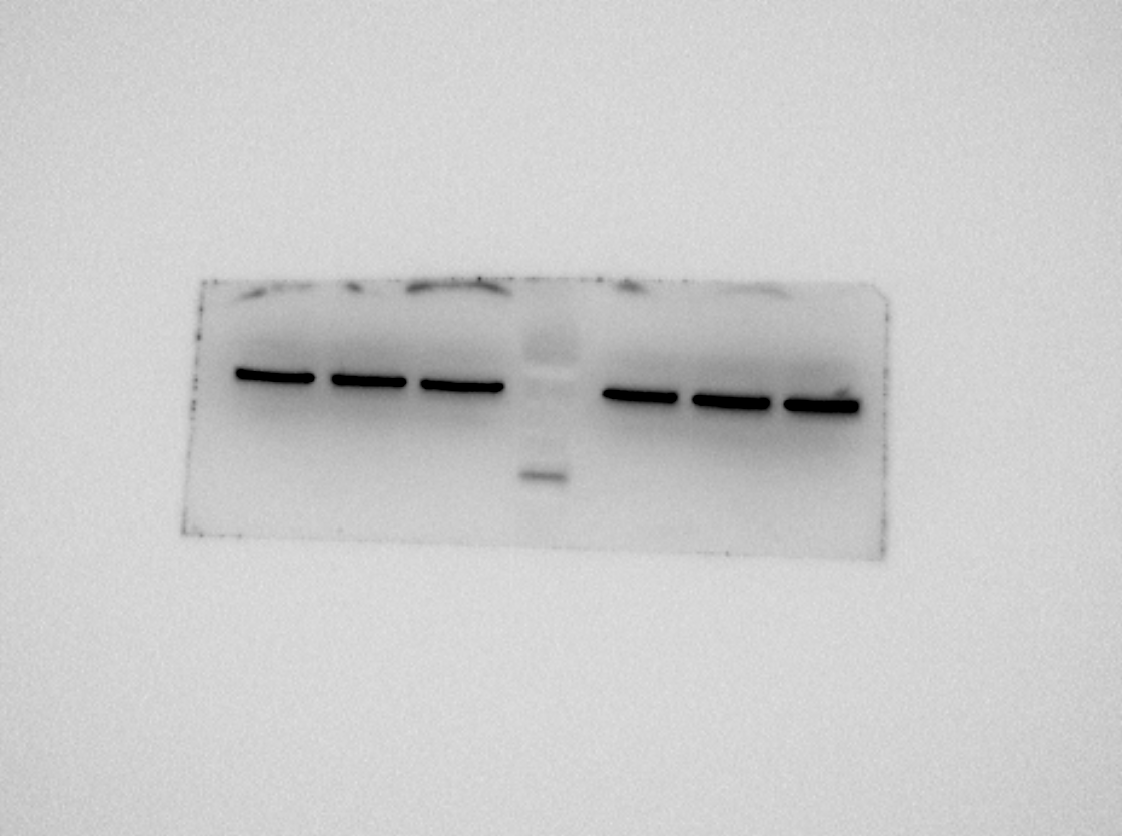

Supplement: Supplementary file 1 [file DataSheet1.zip › Fig4-BECN1.tif]

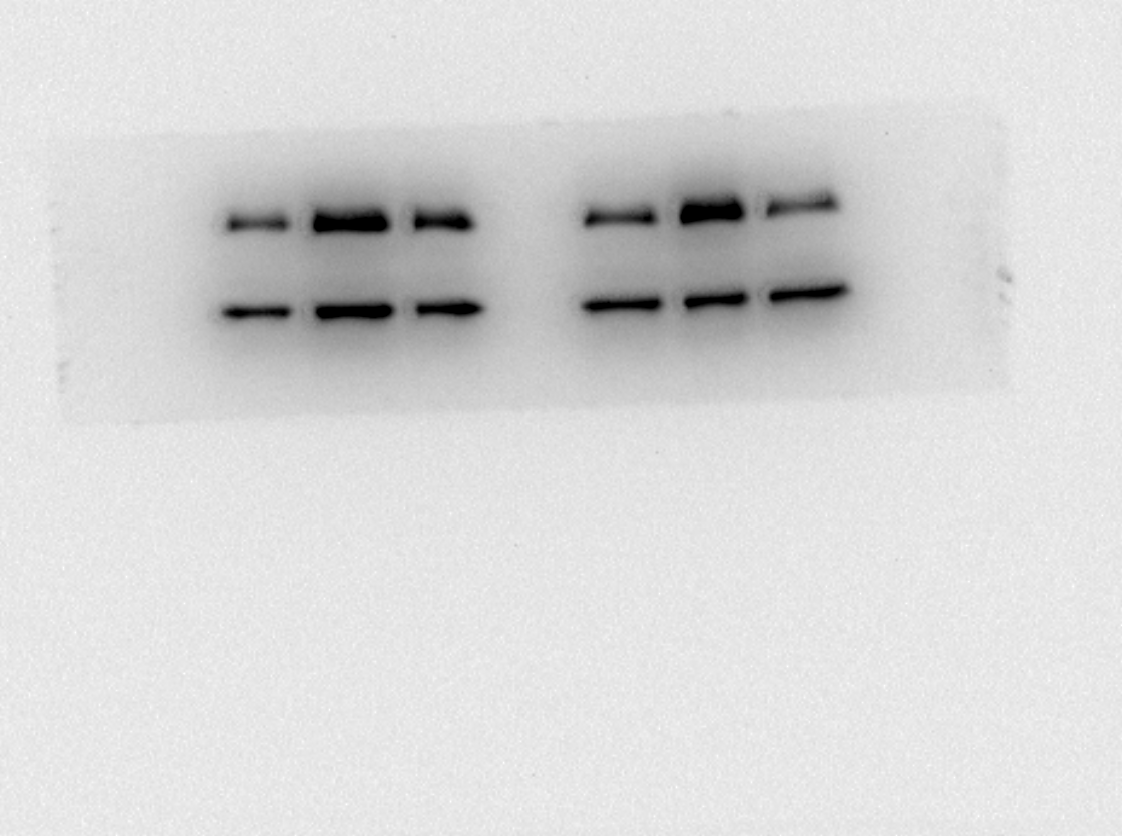

Supplement: Supplementary file 1 [file DataSheet1.zip › Fig4-CAL27-ACSL4-β-actin.tif]

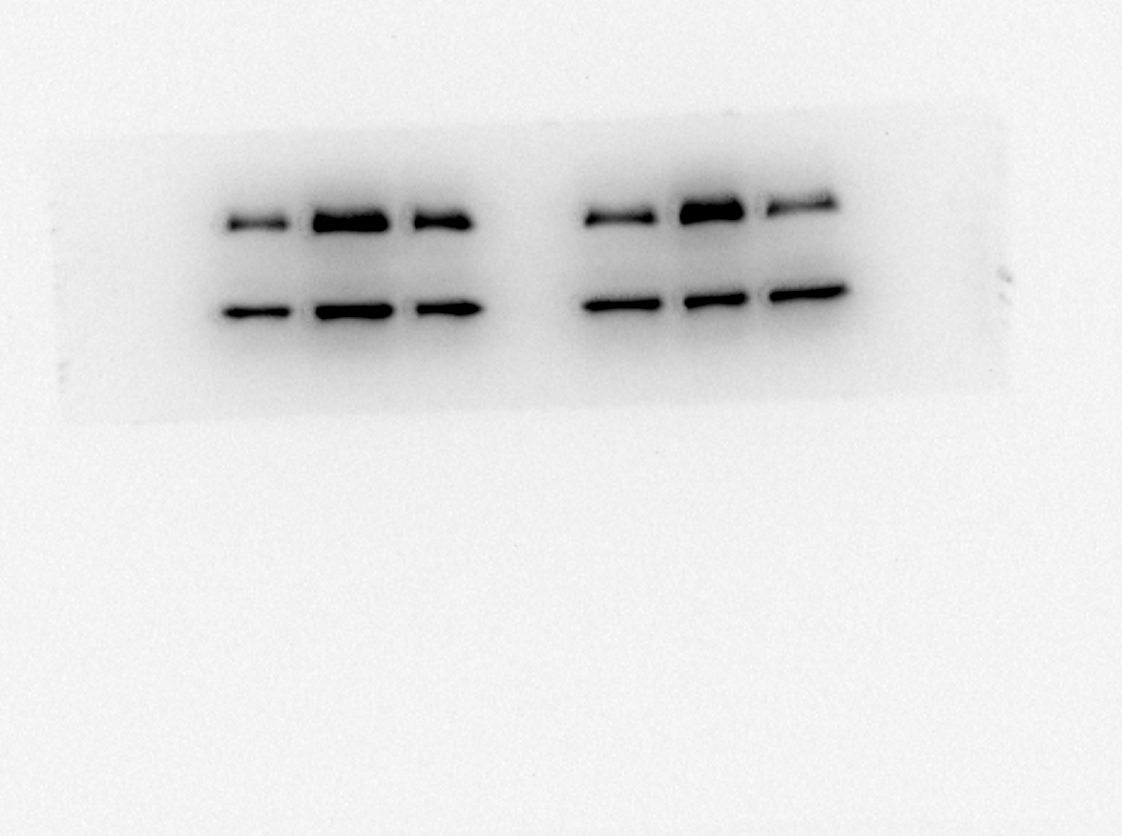

Supplement: Supplementary file 1 [file DataSheet1.zip › Fig4-CAL27-ACSL4.tif]

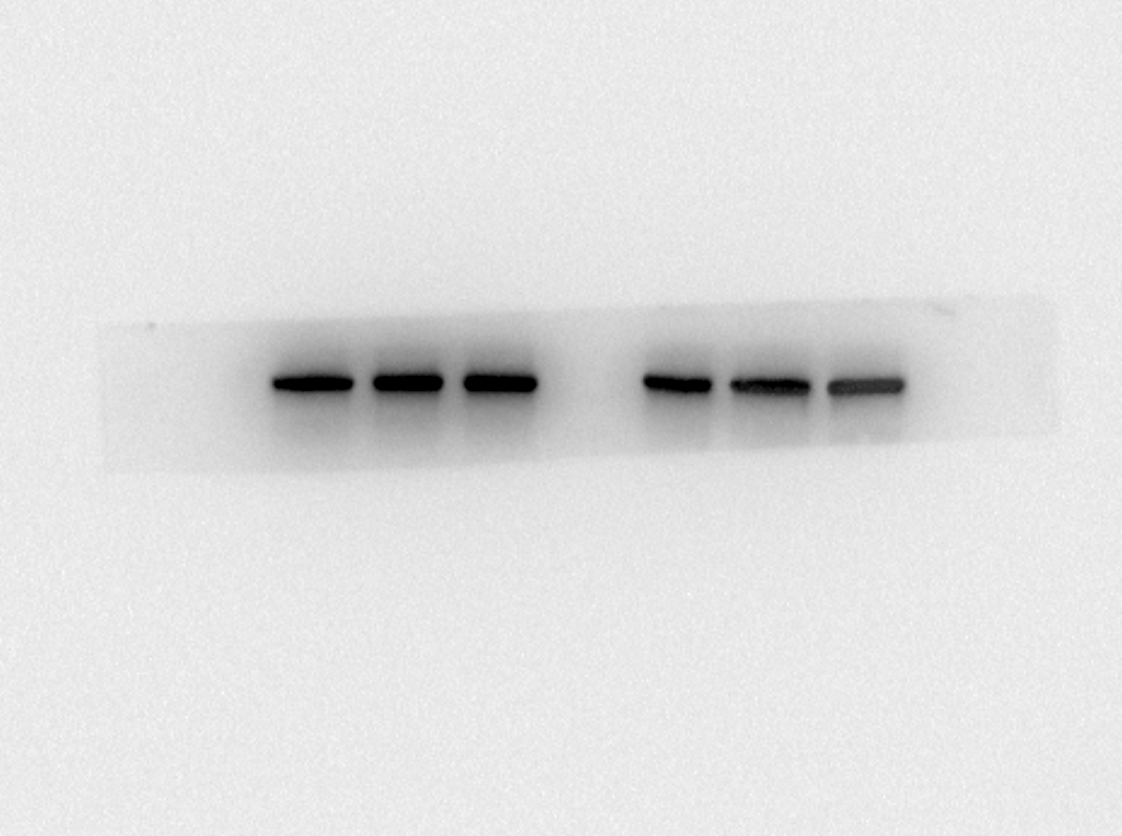

Supplement: Supplementary file 1 [file DataSheet1.zip › Fig4-GPX4-β-actin.tif]

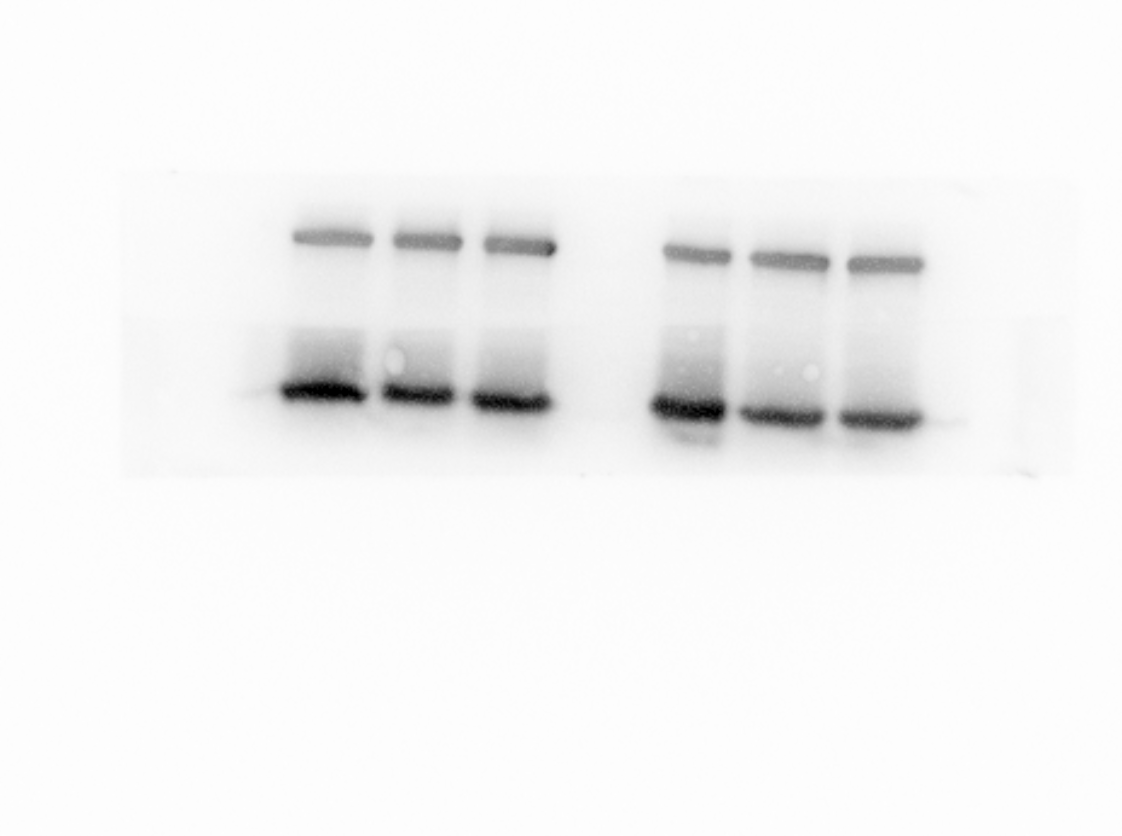

Supplement: Supplementary file 1 [file DataSheet1.zip › Fig4-GPX4.tif]

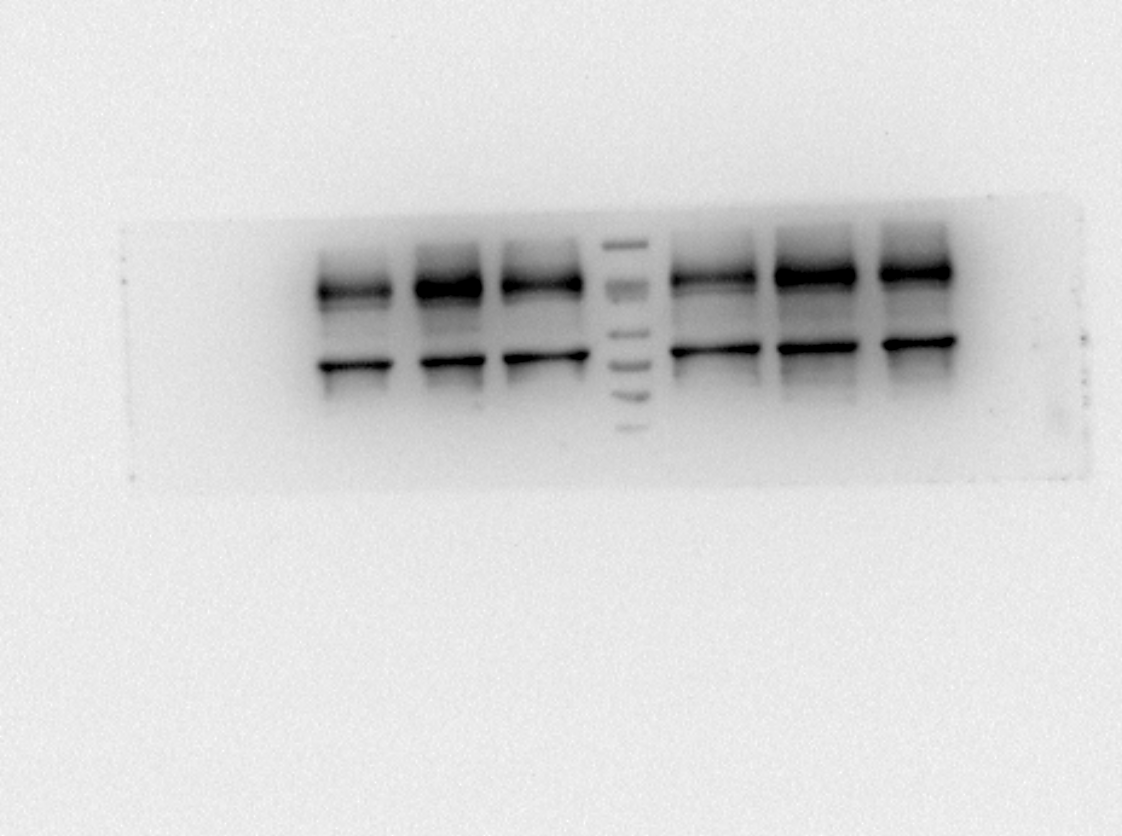

Supplement: Supplementary file 1 [file DataSheet1.zip › Fig4-HN6-ACSL4-β-actin.tif]

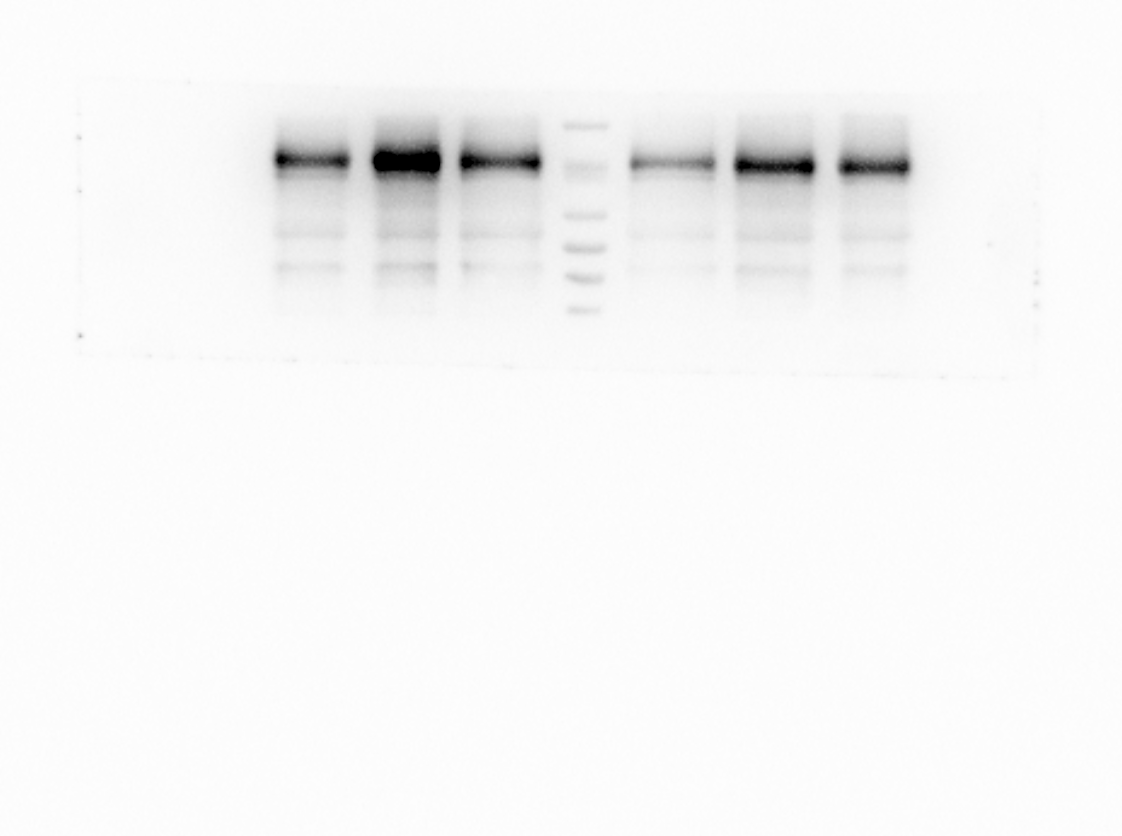

Supplement: Supplementary file 1 [file DataSheet1.zip › Fig4-HN6-ACSL4.tif]

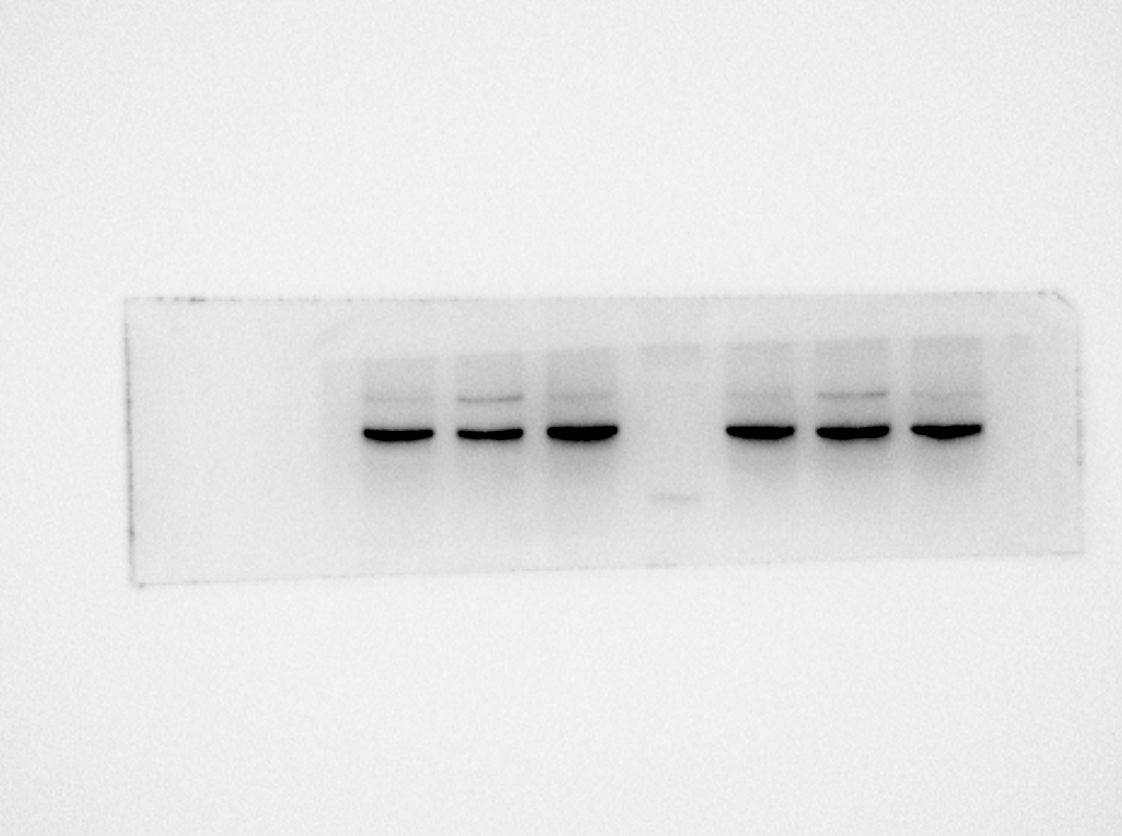

Supplement: Supplementary file 1 [file DataSheet1.zip › Fig4-p-BECN1-β-actin.tif]

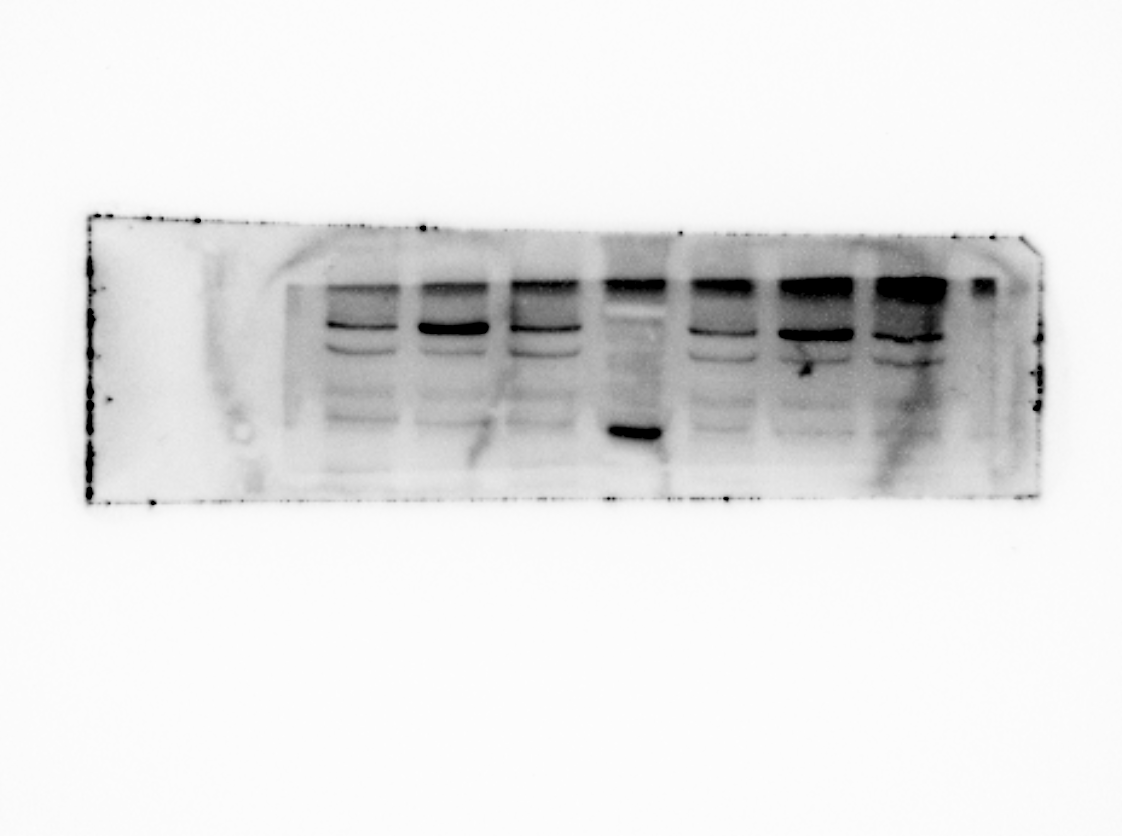

Supplement: Supplementary file 1 [file DataSheet1.zip › Fig4-p-BECN1.tif]
